# Circ_0098181 binds PKM2 to attenuate liver fibrosis

**DOI:** 10.3389/fphar.2025.1517250

**Published:** 2025-04-03

**Authors:** Yuan-Yuan Luo, Ya-Ping Guan, Hong-Fei Zhan, Chun-Yan Sun, Ling-Yan Cai, Ke-Gong Tao, Yong Lin, Xin Zeng

**Affiliations:** ^1^ Department of Gastroenterology, Shanghai East Hospital, Tongji University School of Medicine, Shanghai, China; ^2^ Department of Gastroenterology, Shanghai Changzheng Hospital, Navy Military Medical University, Shanghai, China

**Keywords:** circular RNAs (circRNAs), hepatic fibrosis, adenosine deaminase acting on RNA1 (ADAR1), pyruvate kinase M2, pro-inflammation cytokines

## Abstract

**Background:**

Liver cirrhosis seriously harms human health and fibrosis is the essential pathological process of cirrhosis. Recently, circular RNAs (circRNAs) were found to play critical roles in liver fibrosis, but the key circRNAs and precise mechanisms remained unclear. This study aimed to investigate the effect of circ_0098181 in fibrogenesis and explore its mechanism.

**Methods:**

RNA sequencing was conducted to identify circRNA signatures in human liver cirrhotic tissues. Hepatic stellate cells (HSCs) (including primary rat HSCs, LX2, HSC-T6) and carbon tetrachloride (CCl_4_) induced liver cirrhosis model were used to explore the role of circ_0098181 on HSC activation and liver fibrogenesis *in vitro* and *in vivo*. RNA sequencing, RNA pull-down, mass spectrometry, and RNA immunoprecipitation (RIP) experiments were performed to elucidate the mechanism.

**Results:**

Circ_0098181 was obviously reduced in human fibrotic liver tissues and activated HSCs. Exogenous administration of circ_0098181 blocked the activation, proliferation, and migration of HSCs *in vitro* and mitigated the progression of CCl_4_-induced liver fibrosis *in vivo*. Mechanistically, adenosine deaminase acting on RNA1 (ADAR1) combined with the intronic complementary sequences (ICSs) in the flanking regions, thereby regulating the biogenesis of circ_0098181. RNA sequencing and qRT-PCR revealed the suppression of circ_0098181 on pro-inflammation cytokines expression (TNFα, Fas, Cxcl11, etc.). RNA pull-down, mass spectrometry, and RIP experiments indicated that pyruvate kinase M2 (PKM2) was the direct target of circ_0098181. Circ_0098181 bound to PKM2, restrained its nuclear translocation and phosphorylation.

**Conclusion:**

In conclusion, circ_0098181 exerts a significant anti-fibrotic effect by binding PKM2 to repress its nuclear translocation and inhibiting hepatic inflammation, suggesting the promising therapeutic merit in liver cirrhosis.

## 1 Introduction

Liver cirrhosis, characterized by extracellular matrix (ECM) deposition and persistent inflammation (Parola and Pinzani, 2019), contributes to 2.4% of annual deaths globally (GBD, 2017 Cirrhosis Collaborators, 2020). Currently, cirrhosis is the 11th leading cause of death, responsible for over 1 million fatalities each year (Asrani et al., 2019; Xiao et al., 2019). In China, there are approximately 7 million cirrhotic patients, with 460,000 new cases reported annually (Tapper and Parikh, 2018). Thus, cirrhosis has been a major global health challenge, especially in China. Liver fibrosis, a critical pathological stage in the progression of cirrhosis, is driven by the activation of hepatic stellate cells (HSCs). Therefore, identifying the key factors involved in fibrogenesis and HSCs activation is crucial for developing new therapeutic targets for liver cirrhosis management.

Circular RNAs (circRNAs), with a highly conserved circular structure, are widely present in organisms and play a role in various pathophysiological processes, including the development of major diseases (Misir et al., 2022; Wang et al., 2022; Louis et al., 2022). Due to their unique conformation, stability, and immunogenic properties, circRNAs have significant potentials for biomedical applications. Additionally, several innovative circRNA-based technologies are currently being developed (Zhao et al., 2022; Liu et al., 2022). Recent researches have documented the critical roles of circRNAs in the development of nonalcoholic steatohepatitis (NASH), alcoholic liver disease (ALD), cirrhosis, hepatocellular carcinoma (HCC), and the drug resistance of HCC (Shen et al., 2021; Zeng et al., 2021). Thus far, several studies investigated the circRNAs in liver fibrosis. A sequence of circRNAs, such as circCREBBP, circFBXW4, circPSD3, circ_0070963, and circ_0004018, were reported to regulate HSCs activation and fibrosis (Wang et al., 2023). However, most of these previous studies focused on the sponging mechanism between circRNAs and miRNAs. Recent researches also illustrated some novel and strong roles of circRNAs (Huang et al., 2020). For example, circRNAs could directly combine with proteins, and regulate the biological function or mediate the degradation of proteins (Huang et al., 2020; Zhou et al., 2020). Additionally, with an internal ribosome entry site (IRES) and an open reading frame (ORF), some circRNAs could even translate specific proteins (Lei et al., 2020). To our knowledge, there are few studies focused on the direct combination between circRNAs and proteins in fibrogenesis.

According to the recommended experimental scheme for understanding the cellular roles of circRNAs (Liu and Chen, 2022), the mechanism and regulation of cyclization and biogenesis should be involved in circRNAs researches. Generally, circRNAs are generated from the pre-mRNAs of gene exons through back-splicing depending on the spliceosome. The back-splicing was regulated by intronic complementary sequences (ICSs) in the flanking regions, and proteins might bind to the flanking segments. Some specific proteins, such as adenosine deaminase acting on RNA 1(ADAR1), quaking (QKI), FUS, DXH9, etc., could bind the flanking introns of circle-forming exons and influence the intronic ICS pairs, leading to the enhancement or impairment of circRNAs generating (Huang et al., 2020). At present, the biogenesis and regulation manner of multiple circRNAs in fibrosis is unexplored.

With this study, we screened the distinctive circRNAs in human liver cirrhotic tissues by RNA sequencing and verified the downregulation of circ_0098181 in activated HSCs. Circ_0098181 was then delivered into primary rats HSCs, HSCs lines, and carbon tetrachloride (CCl_4_) induced fibrotic rats to investigate the role of circ_0098181 on HSCs activation and liver fibrosis. Moreover, we aimed to clarify the biogenesis manner and the mechanisms of circ_0098181 in fibrogenesis.

## 2 Materials and methods

### 2.1 Patients and samples

Human liver tissue samples (5 cirrhotic liver tissues, 5 HCC tissues, and 5 normal liver tissues) were obtained from patients who underwent liver transplantation or surgical resection at the Eastern Hepatobiliary Surgery Hospital (Shanghai, China). Five normal liver tissues were collected from patients with hemangioma. Five cirrhotic liver tissues were obtained from patients with decompensated cirrhosis who underwent liver transplantation. The clinical data are provided in Supplementary Table S1. Written informed consent was obtained from all patients. The study was approved by the Medical Ethics Committee of the Eastern Hepatobiliary Surgery Hospital.

### 2.2 Cell culture and rat primary HSCs isolation

LX2 cells and HSC-T6 were obtained from the Type Culture Collection of the Chinese Academy of Sciences (Shanghai, China) and cultured in Dulbecco’s modified Eagle’s medium (DMEM) with 2%–10% fetal bovine serum (FBS) and 1% penicillin–streptomycin. To confirm the reduction of circ_0098181 in activated HSCs, LX2 were stimulated with 10 ng/mL TGF-β1 (PeproTech, 100-21C) for 48 h.

To determine the decrease level of circ_0098181 in liver fibrogenensis, we also measured the expression of circ_0098181 in activated rat primary HSCs on the 14th day after HSCs isolation compared with the quiescent HSCs on the second day. Sprague-Dawley (SD) rats, weighing about 200 g, were purchased from the Experimental Animal Center of Naval Medical University. As previously described (Friedman and Roll, 1987), liver cells were isolated using a mixture of 0.5 mg/mL IV collagenase (C5138, Sigma-Aldrich), 0.5 mg/mL Pronase E (Roche), and 1% DNase. HSCs were then separated by gradient centrifugation with 40% and 60% Percoll (P4937, Sigma-Aldrich). The quiescent HSCs were cultured in DMEM with 10% FBS and 1% penicillin–streptomycin. After 14 days in culture, these quiescent rat HSCs became activated, exhibiting high expression of α-SMA. The expression of circ_0098181 was also determined in auto-activated primary rat HSCs.

### 2.3 Plasmid and cell transfection

Plasmids expressing has_circ_0098181 and the control plasmids (NC) were purchased from Geneseed Biotech (Guangzhou, China). Due to the high homology (91.93%) between the human and rat sequences of circ_0098181, plasmid contained has_circ_0098181 sequence was transfected into TGF-β1 (2.5 ng/mL) pre-treated LX2 cells, HSC-T6 cells, and auto-activated primary rat HSCs with lipofectamine 2000 (Lipofectamine 2000; Thermo Fisher Scientific, America). After 48 h, cells were extracted to perform qRT-PCR and Western blotting. Different primers designed for human and rat circ_0098181 sequences were used to detect the level of circ_0098181 among different species.

### 2.4 CCK-8 assay and transwell experiment

CCK-8 assay was performed to explore the roles of circ_0098181 on HSCs proliferation. Approximately 3,000 cells were seeded into each well of a 96-well plate, followed by transfection with circ_0098181 plasmid at the next day. Cell proliferation was then measured daily at 450 nm using the Cell Counting Kit-8 (Dojindo, Tokyo, Japan). Due to the slow proliferation of rat primary HSCs, the medium was refreshed daily to support cell growth.

Additionally, transwell assays were conducted to assess cell migration. Cells pre-transfected with circ_0098181 plasmids were seeded into transwell inserts equipped with 8.0-μm-pore filters (BD, NJ). After 72 h, cell migration was evaluated by counting the cells on the underside of the inserts using ImageJ software (ImageJ 1.x, National Institutes of Health, USA).

### 2.5 RNA extraction and qRT-PCR

Total RNA was extracted from cells and tissues with Trizol Reagent (TaKaRa, Tokyo, Japan). cDNA was synthesized by reverse transcription and qRT-PCR was performed with PrimeScript RT Master Mix and TB Green Premix EX Taq (TaKaRa, Tokyo, Japan). The primers were designed by Primer3 Plus (https://www.bioinformatics.nl/cgi-bin/primer3plus). The detailed primer sequences are shown in Supplementary Table S2.

### 2.6 Western blotting assays and cytoplasmic and nuclear fractionation

The proteins were extracted with lysis from cells or tissues, and the supernatant was centrifuged to perform further quantification. Minute (TM) Cytoplasmic and Nuclear Fractionation Kit (SC-003, Invent) was used to extract cytoplasmic protein and nuclear protein, and Western blotting assays were performed according to the manufacturer’s instructions. After SDS-PAGE electrophoresis, proteins were transferred to a PVDF membrane. Then the membrane was blocked in 5% milk and incubated with the primary antibody overnight, followed by 1 h’s incubation with the appropriate secondary antibody. Signals were scanned with the Odyssey infrared imaging scanner (LI-COR Odyssey system). The antibodies are listed in Supplementary Table S3.

### 2.7 Immunofluorescence staining

For immunofluorescence staining, cells were first fixed with 4% PFA for 10 min, then permeabilized with 0.1% Triton X-100 and blocked with 5% BSA. The cells were incubated with the primary antibody overnight, followed by incubation with the secondary antibody for 1 h the next day. Nuclei were stained with DAPI, and the signals were observed using a confocal microscopy.

### 2.8 Fluorescence *in situ* hybridization

Fluorescence *in situ* hybridization (FISH) was performed to explore the location of circ_0098181 in HSCs according to the manufacturer’s recommended methods. The FISH probe for circ_0098181 and the positive control of cytoplasm (18S probe) were synthesized by Ribobio Company (Guangzhou, China). In brief, the cells were fixed with 4% Paraformaldehyde (PFA) and perforated with 0.5% Triton X-100. Then cells were incubated with a pre-hybridization solution for 30 min, and hybridized with the hybridization solution (including probes, 20 μM) at 37°C overnight. On the next day, cells were washed with 4 × SSC, 2 × SSC, and 1 × SSC three times each in turn. Finally, DAPI was used to stain the nucleus and the signals were detected by a confocal microscopy.

### 2.9 Animal models and treatment

To explore the roles of circ_0098181 *in vivo*, a rat fibrotic model was constructed. SD rats, weighing about 150–160 g, were intraperitoneally injected with CCl_4_ (0.5 mL/kg, CCl_4_ = 1:3) twice a week for total 6 weeks to induce liver fibrosis, while the controls were intraperitoneally injected with Oil. Given that adeno-associated virus-8 (AAV8), a hepatotropic virus, could specifically target hepatocytes, we selected adenovirus to overexpress circ_0098181, enabling effective infection of hepatic stellate cells (HSCs). An adenovirus carrying the rat circ_0098181 sequence (Ad-circ_0098181) and a control virus (Ad-NC) were constructed by Genechem Co. Ltd (Shanghai, China). Twenty SD rats were randomly assigned to four groups: Oil + Ad-NC, Oil + Ad-circ_0098181, CCl_4_ + Ad-NC, and CCl_4_ + Ad-circ_0098181. At the fourth week after CCl_4_ or oil administration, Ad-circ_0098181 (1 × 10^10^ pfu) or Ad-NC (1 × 10^10^ pfu) was injected into the tail vein. The rats were sacrificed 2 weeks after adenovirus delivery.

Serum was collected for ALT and AST measurements, and liver tissues were harvested for further analysis, including HE, Sirius Red, and immunohistochemistry (IHC) staining for Col1a1 and α-SMA. The remaining liver tissues were used for RNA and protein extraction for additional validation.

### 2.10 Histology and immunohistochemistry

Liver paraffin sections (4 μm) were deparaffinized and hydrated to remove the paraffin and allow staining reagents to penetrate. HE, Sirius Red, and immunohistochemistry staining were performed as previously described (Huang et al., 2023). Images of HE, Sirius Red, and immunohistochemistry staining were captured using an Olympus BX43F microscope.

### 2.11 RNA sequencing and bioinformatics analysis

To investigate the downstream genes and pathways of circ_0098181, HSC-T6 cells treated with either circ_0098181 (n = 3) or NC (n = 3) plasmids were used to perform RNA sequencing by Shanghai Biotechnology Corporation (China). Briefly, total RNA was extracted using Trizol reagent (Invitrogen). RNA concentration and purity were measured using a NanoDrop 2000 system (Thermo Fisher Scientific, Wilmington, DE, USA). Sequencing was performed on NovaSeq6000 (Illumina) using PE150 mode. All the downstream analyses were conducted on high-quality clean data. HISAT2 tools software (version:2.0.4) was used to map to the reference genome. Finally, differentially expressed genes (DEGs) were identified using the edgeR R package. Multiple testing correction was applied using the Benjamini–Hochberg method to control the false discovery rate. Genes with an adjusted p value < 0.05 and an absolute value of log2 (Fold change) > 1 were considered to be differentially expressed. The ClusterProfiler R package was used to identify significantly enriched Kyoto Encyclopedia of Genes and Genomes (KEGG) pathways compared to the entire genome background. Gene Set Enrichment Analysis (GSEA) was performed using GSEA software 3.0. Additionally, protein-protein interaction (PPI) network analysis was performed to highlight relevant and potential pathways.

### 2.12 SiRNA design and plasmids contained circ_0098181 sequence and its flanking sequence

SiRNAs targeting ADAR1 and QKI within rat genomes were synthesized by General Biology (Anhui, China), with the sequences provided in Supplementary Table S4. Based on the silencing efficiency of these siRNAs, shRNA targeting ADAR1 (shRNA-ADAR1) was designed. The plasmids containing the rat circ_0098181 sequence along with its flanking regions were supplied by Shanghai Calm Biotechnology Co., Ltd., with detailed information on the flanking sequences available in Supplementary Table S5.

### 2.13 The binding segment between PKM2 and circ_0098181

To determine the binding region of PKM2, the PKM2 gene was divided into three equal segments. Plasmids containing these three segments, along with the full-length PKM2 (details in Supplementary Table S6), were constructed and transfected into HSC-T6 cells for RNA immunoprecipitation (RIP) assays. Similarly, to identify the binding sites of circ_0098181, the sequence of circ_0098181 was divided into three segments, and primers were designed based on each segment for RIP experiments. The primers for these segments are listed in Supplementary Table S2.

### 2.14 RNA pull-down, mass spectrum, and RIP

For RNA pull-down, the circ_0098181 positive probe (5′-biotin CTG​GTC​GCT​TGG​AAG​ACA​TCC​TTC​CGG​CTC​GTT​CTT​GAT​C-3′) and negative probe (5′-biotin GAT​CAA​GAA​CGA​GCC​GGA​AGG​ATG​TCT​TCC​AAG​CGA​CCA​G-3′) were provided by Cloud Biotech (Shanghai, China). After proteins were pulled down with the RNA probes in HSC-T6 cells, the eluted proteins were analyzed with mass spectrum. Easy nLC 1000 system (ThermoFisher Scientific) was used for liquid chromatography separation of peptide segments, and mass spectrometry analysis was performed with a Q-Exactive master spectrometer (ThermoFisher Scientific). RIP experiments were taken in HSC-T6 cells (about 1 × 107) with RNA Immunoprecipitation Kit (Geneseed Biotech, Guangzhou, China). After digestion, the cells were dissociated with lysis buffer. The supernatant was hybridized with magnetic beads that had been bound to PKM2 or rabbit IgG antibodies. Total RNA which bound proteins were eluted for further qRT-PCR.

### 2.15 Statistical analysis

Continuous parameters were presented as the mean ± standard deviation. Categorical variables were expressed as numbers and percentages or frequencies. For the statistical analysis, one-way analysis of variance (ANOVA) was determined for the analysis of multiple groups involving a single variable. Differences in categorical variables between groups were assessed using the Chi-square test. All statistical analyses were performed using R software version 4.4.1 (R Foundation for Statistical Computing, Vienna, Austria).

Experimental data were collected and analyzed by SPSS V23.0 or Prism8 software (GraphPad Software, La Jolla, CA). The transwell, IHC, and Sirus red staining were analyzed by ImageJ software. Data were presented as the mean ± standard deviation. For datasets that conformed to a normal distribution, a Student’s t-test was utilized to compare two datasets. In cases where the data did not follow a normal distribution, the Mann-Whitney U test was applied. When analyzing multiple groups with a single variable, one-way ANOVA was employed. For comparing more than two groups with multiple variables, two-way ANOVA was used. p-value <0.05 was reckoned statistically significant (*p < 0.05, **p < 0.01, ***p < 0.001).

## 3 Results

### 3.1 Circ_0098181 is downregulated in the liver cirrhosis tissues and activated HSCs

**FIGURE 1:**
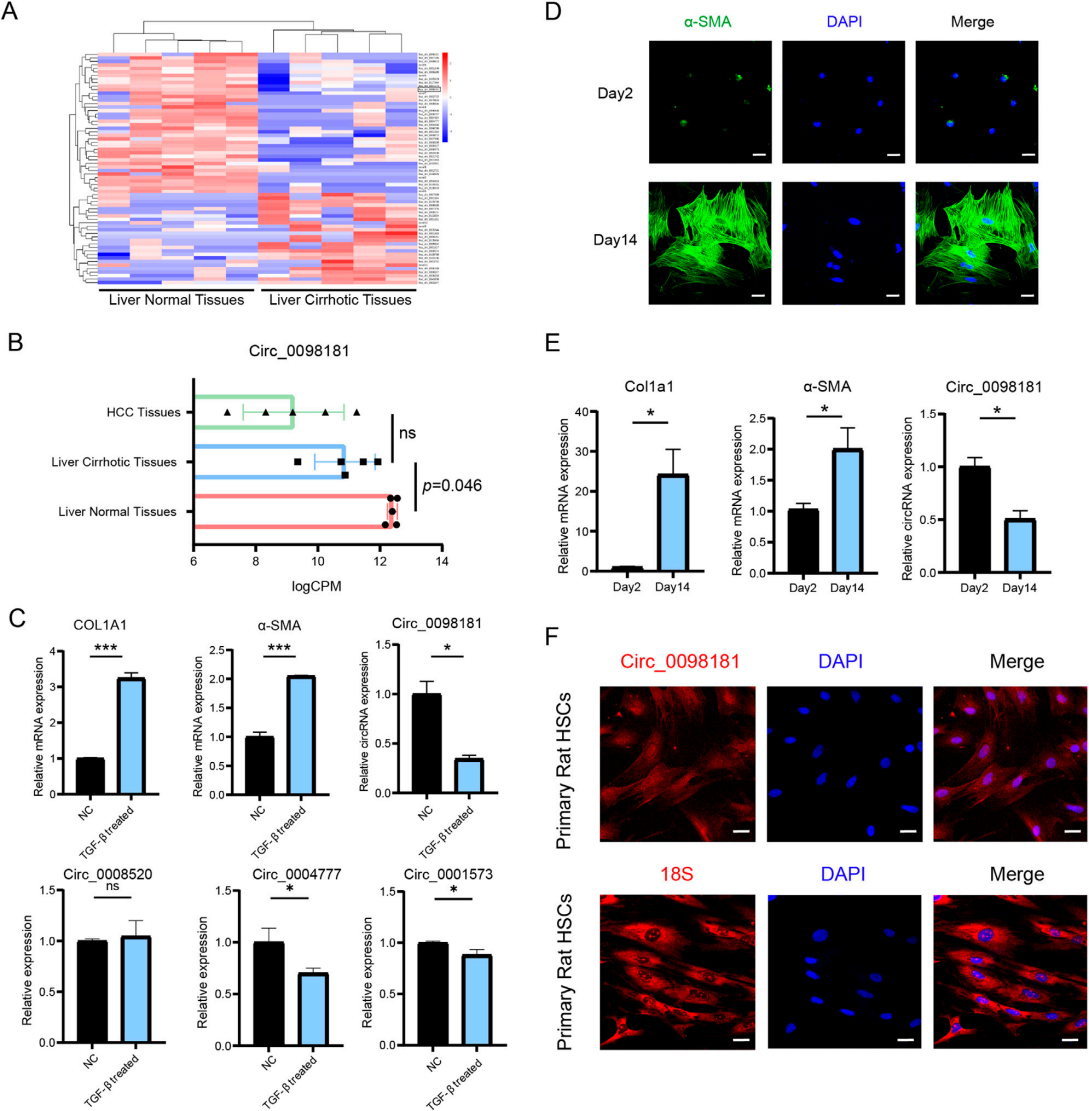
Circ_0098181 decreases in liver fibrosis. **(A)** The specific circRNAs profiles were screened by RNA sequencing between normal and fibrotic human liver tissues (n = 5). **(B)** Expression of circ_0098181 on HCC tissues, liver fibrotic tissues and normal liver tissues (n = 5). **(C)** The expressions of circRNAs, COL1A1 and α-SMA in activated LX2 cells with TGF-β1 treatment. **(D)** Activated primary rat HSCs on Day 14 showed a high expression of α-SMA. Scale bars, 10 μm. **(E)** Upregulation of Col1a1 and α-SMA, and downregulation of circ_0098181 on Day 14 of primary rat HSCs compared with Day 2. **(F)** Circ_0098181 was located in the cytoplasm. 18S was the positive cytoplasmic control. Scale bars, 10 μm *p < 0.05, **p < 0.01, ***p < 0.001.

The specific circRNAs profiles were screened by RNA sequencing in five paired human cirrhotic liver tissues and normal samples. Based on fold-change, there were 26 upregulated and 41 downregulated circRNAs in cirrhotic tissues, including circ_0000131, circ_0098181, circ_0036353, circ_0008520, circ_0004777, and so on (Figure 1A). Among the identified circRNAs, circ_0098181 exhibited a significant reduction in cirrhotic samples compared with that in normal tissues (p < 0.05). Further analysis demonstrated that circ_0098181 levels were highest in normal tissues, decreased in cirrhotic tissues, and were lowest in HCC tissues (Figure 1B), indicating a strong correlation between declining circ_0098181 expression and the progression of liver damage. Consistent with the data in tissues, our observations also revealed that circ_0098181 was most reduced in TGF-β1 treated LX2 cells compared with circ_0008520, circ_0004777, and circ_0001573, as well as in auto-activated primary rat HSCs, accompanied by increased expression of Col1a1 and α-SMA. (the marker of activation of HSCs) (Figures 1C–E; Supplementary Figure S1A). Furthermore, the FISH probe illustrated the enrichment of circ_0098181 in the cytoplasm of primary rat HSCs and activated LX2 cells (Figure 1F; Supplementary Figure S1B).

### 3.2 Circ_0098181 inhibits the activation and proliferation of HSCs

**FIGURE 2:**
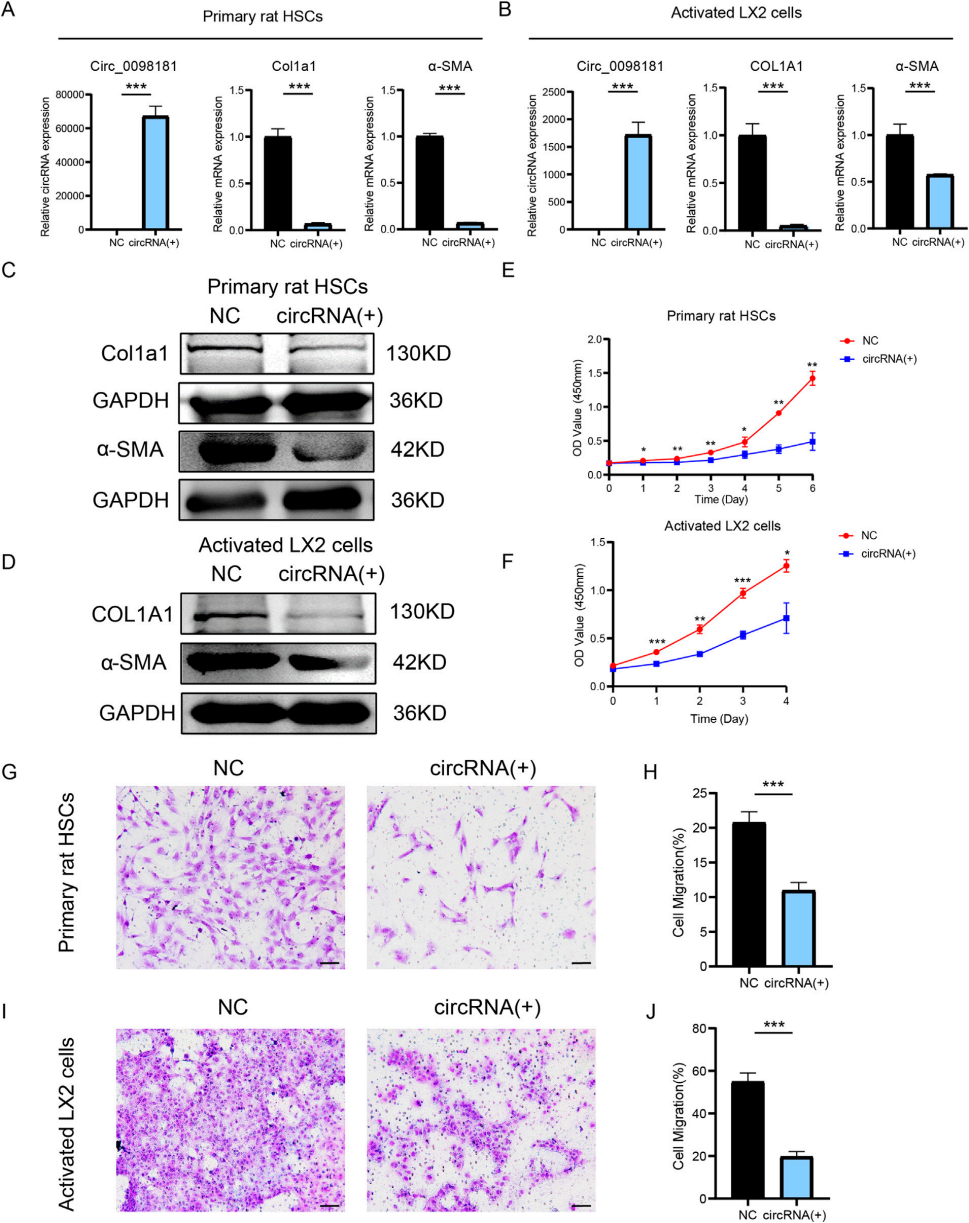
Circ_0098181 suppresses the activation, proliferation, migration of HSCs. **(A, B)** qRT-PCR illustrated the inhibition of Col1a1 and α-SMA after circ_0098181 delivery in primary rat HSCs and LX2. **(C, D)** Western blotting results demonstrated that overexpressing circ_0098181 could suppressed Col1a1 and α-SMA protein level in primary rat HSCs and LX2. **(E, F)** Upregulating circ_0098181 restrained the proliferation of HSCs. The primary rat HSCs in the two groups were stimulated with TGF-β1. **(G–J)** Enhancing circ_0098181 mitigated the migration of HSCs. Scale bars, 50 μm *p < 0.05, **p < 0.01, ***p < 0.001.

Given the high homology (91.93%) between the human and rat sequences of circ_0098181, plasmids carrying has_circ_0098181 were transfected into primary rat HSCs and activated LX2 to further explore the biological function of circ_0098181 (Figures 2A, B). qRT-PCR and Western blotting demonstrated that circ_0098181 remarkably suppressed the mRNA and protein levels of Col1a1 and α-SMA (Figures 2A–D). CCK-8 assay verified the suppression of circ_0098181 on HSCs proliferation in both primary HSCs and LX2 (Figures 2E, F). Furthermore, transwell experiments documented that circ_0098181 obviously inhibited the migration of HSCs (Figures 2G–J). Additionally, circ_0098181 also reduced the expression of Col1a1 and α-SMA, and suppressed the proliferation and migration of HSC-T6 (Supplementary Figures S2A-D). All these findings highlight the evident inhibitory effect of circ_0098181 on HSCs activation and proliferation *in vitro*, suggesting its potential as a therapeutic target for liver fibrosis.

### 3.3 Circ_0098181 ameliorates liver fibrosis *in vivo*

**FIGURE 3:**
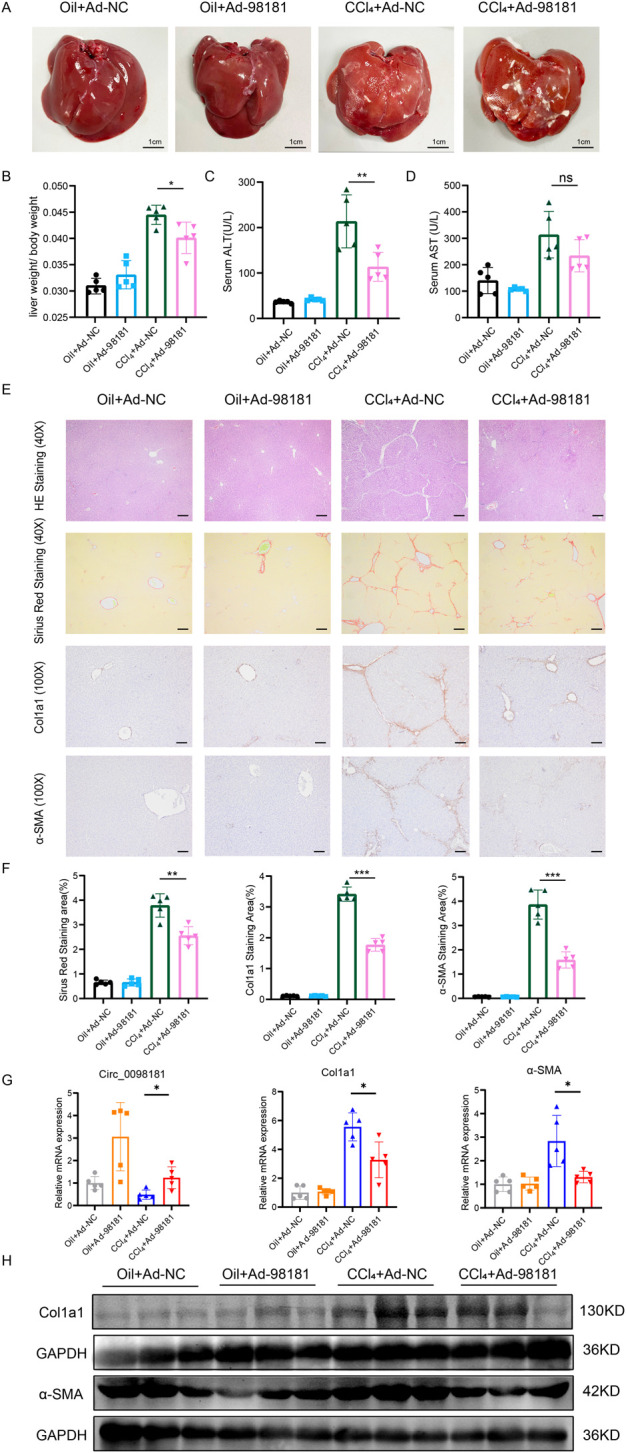
Circ_0098181 mitigates the progression of rat liver fibrosis. **(A)** The general macroscopic view of the rat livers (n = 5). Scale bars, 1 cm. **(B–D)** The liver/body weight ratio, serum ALT(U/L), and serum AST(U/L) levels in four groups. **(E)** HE and Sirus Red staining demonstrated that the inflammation and collagen deposition, induced by CCl_4_, could be alleviated by circ_0098181 adenovirus. Scale bars (40X), 20 μm. The results of immunohistochemistry staining vindicated that upregulation of circ_0098181 suppressed Col1a1 and α-SMA. Scale bars (100X), 50 μm. **(F)** Sirius red, immunohistochemical staining were analyzed with Image J. **(G)** mRNA expression levels of circ_0098181, Col1a1 and α-SMA in rat liver tissues. **(H)** Protein expression levels of Col1a1 and α-SMA in rat liver tissues. *p < 0.05, **p < 0.01, ***p < 0.001.

Adenovirus carrying circ_0098181 was injected into CCl_4_-induced rat fibrosis models to explore the effect of circ_0098181 on fibrogenesis. In the sixth week after intraperitoneal injection of CCl_4_, the livers were enlargement, and the surface became rough and granular, indicating the significant manifestations of liver fibrosis induced by CCl_4_. In contrast, rats treated with Ad-circ_0098181 (CCl_4_+Ad-circ_0098181 group) displayed more normal liver morphology, with a smoother surface, compared to those treated with the control virus (CCl_4_+Ad-NC group) (Figure 3A). In addition, the liver-to-body weight ratio and serum ALT levels were significantly lower in the CCl_4_+Ad-circ_0098181 group compared to the model group (CCl_4_+Ad-NC) (both p < 0.05) (Figures 3B–D). However, there were no significant differences in serum AST levels between the two groups. Exogenous administration of circ_0098181 notably reduced the severity of fibrosis and collagen deposition, as evidenced by HE and Sirius Red staining (Figures 3E, F). Additionally, immunofluorescence staining, qRT-PCR, and Western blotting revealed decreased expression of Col1a1 and α-SMA in the circ_0098181-treated rats (CCl_4_+Ad-circ_0098181 group) compared to the CCl_4_+Ad-NC group (Figures 3G, H). These results demonstrate the strong anti-fibrotic effects of circ_0098181 *in vivo*.

### 3.4 ADAR1 combines with ICSs in the flanking introns and regulates the biogenesis of circ_0098181

**FIGURE 4:**
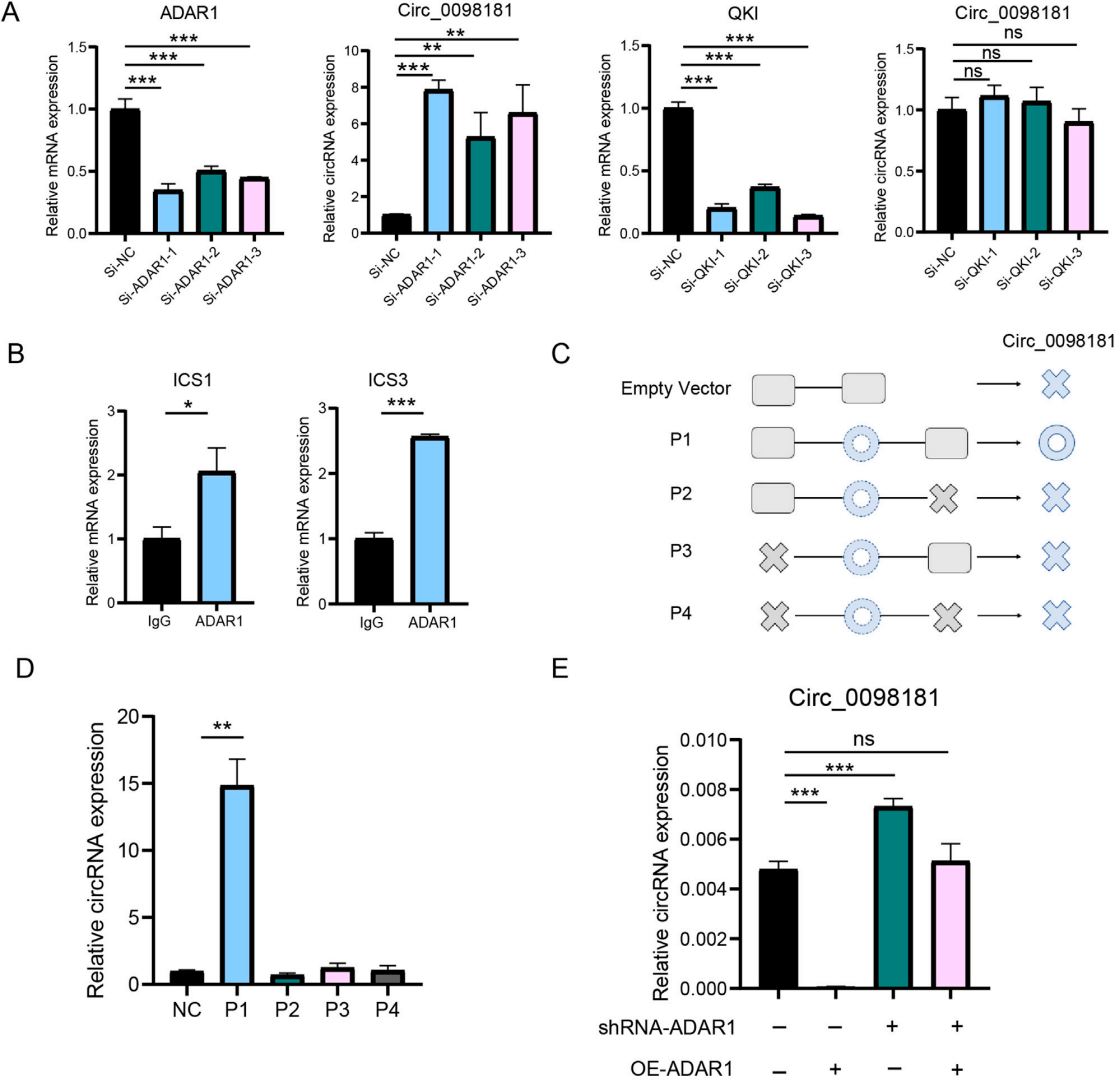
ADAR1 mediates the biogenesis of circ_0098181. **(A)** SiRNA of ADAR1, QKI and control was transfected to HSC-T6 cells, and qRT-PCR documented that only ADAR1 could regulate the expression of circ_0098181. **(B)** RIP experiment verified that ADAR1 directly bound ICS1 and ICS3. **(C)** The design for plasmids of the flanking sequence of circ_0098181. P1: containing whole circ_0098181, ICS1 and ICS3. P2: containing ICS1 and full length of circ_0098181. P3: containing ICS3 and whole circ_0098181. P4: only containing whole circ_0098181. NC: pCDNA3.1 empty plasmid. **(D)** Only P1 could produce more circ_0098181. **(E)** The inhibition of shRNA-ADAR1 on circ_0098181 could be reversed by overexpressing ADAR1. *p < 0.05, **p < 0.01, ***p < 0.001.

Previous studies have documented that circRNAs are produced from the pre-mRNAs of gene exons by back-splicing. According to the data from circBase (http://www.circbase.org/), circ_0098181 is derived from exons 2 and 3 of the SOX5 gene. Hereby, we elucidated the synthesis regulation of circ_0098181 in liver fibrogenesis. RNA binding protein-driven cyclization was one of the important approaches to generate circRNAs. ADAR1 and QKI, two major RNA binding proteins involved in circRNA cyclization, could competitively bind to ICSs in the flanking intron of circRNAs and regulate the biogenesis of circRNAs (Ivanov et al., 2015; Xi et al., 2022). To identify the key RNA-binding protein involved in circ_0098181 cyclization, we synthesized siRNAs targeting ADAR1 and QKI, and transfected them into HSC-T6 cells. The expression of ADAR1 and QKI was successfully knocked down by their respective siRNAs (Figure 4A). However, an increase in circ_0098181 levels was only observed following si-ADAR1 transfection. Additionally, sequence alignment of intron 1 and intron 3 of the SOX5 gene revealed highly reverse complementary sequences (approximately 80%) in the flanking introns of circ_0098181(Supplementary Figures S3A, B), rather than paired Alu sequences (Zhang et al., 2014). Thus, we hypothesized that ADAR1 might bind to the ICSs in the flanking regions, thereby regulating the synthesis of circ_0098181. We termed the complementary sequences in intron 1 and intron 3 of the SOX5 gene as ICS1 (reverse complementary sequences in intron 1) and ICS3 (reverse complementary sequences in intron 3), respectively. Primers for ICS1 and ICS3 were then designed to conduct RIP experiments. The results demonstrated that ADAR1 could bind to both ICS1 and ICS3 (Figure 4B). Furthermore, plasmids containing the full circ_0098181 sequence, along with ICS1 and ICS3 (P1), and plasmids with missing ICS1 or ICS3 (P2-P4), were constructed and transfected into HSC-T6 cells (Figure 4C; Supplementary Table S5). As shown in Figure 4D, circ_0098181 was significantly upregulated in HSC-T6 cells transfected with the P1 plasmid, while plasmids P2-P4 had no effect on circ_0098181 levels. Moreover, to confirm the ADAR1-dependent cyclization, a plasmid overexpressing ADAR1 was introduced into HSC-T6 cells transfected with shRNA-ADAR1. As expected, the inhibition of ADAR1 prevented the reduction of circ_0098181 induced by ADAR1 overexpression (Figure 4E). These findings suggest that ADAR1 suppresses the biogenesis of circ_0098181 by disrupting the intronic ISC pairs.

### 3.5 Circ_0098181 suppresses the inflammation of the liver

**FIGURE 5:**
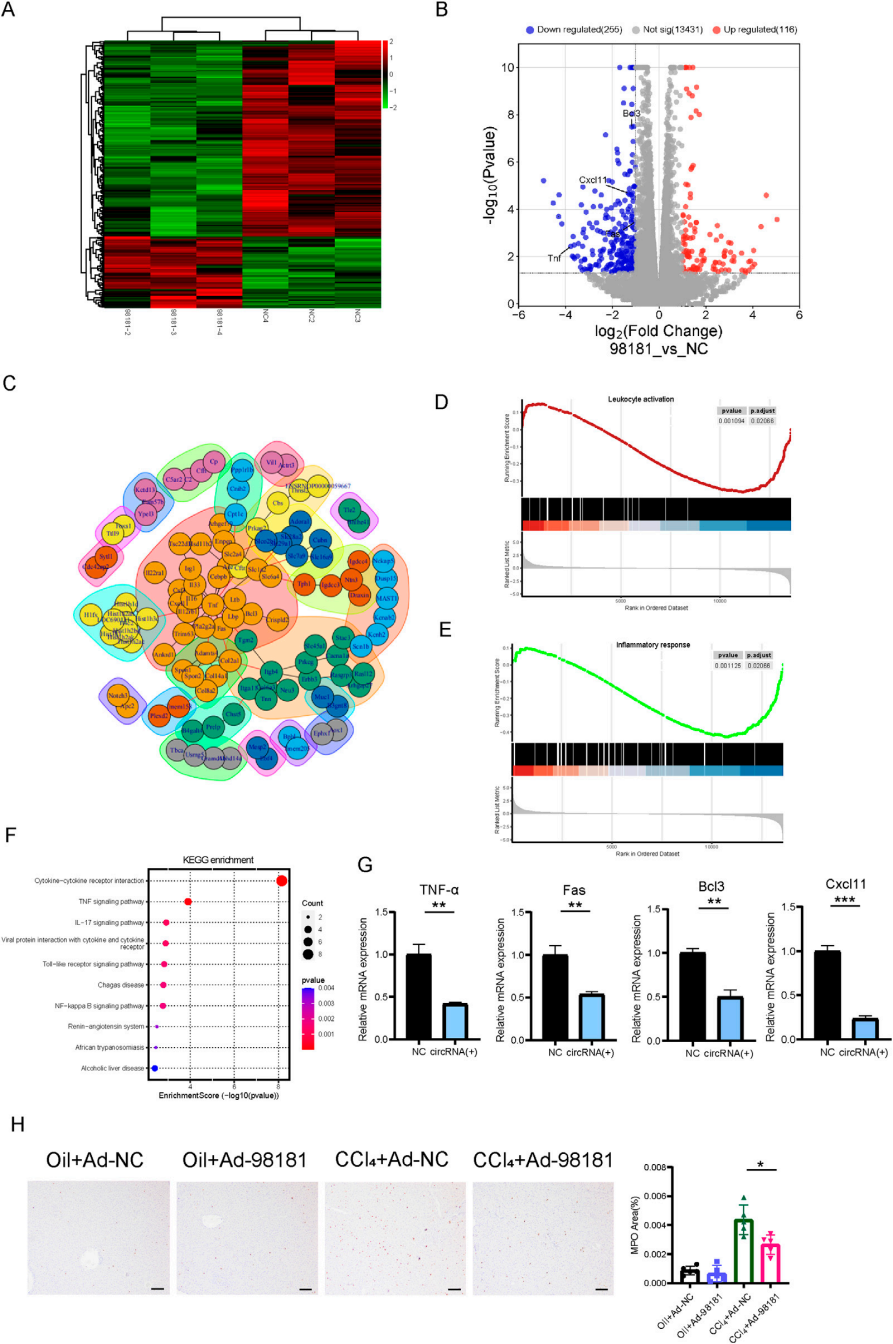
Circ_0098181 suppresses the inflammation of the liver. **(A)** RNA sequencing volcano plot of differential genes in HSC-T6 cells treated with circ_0098181 (n = 3) and control (n = 3) plasmids. **(B)** Volcano map of differentially expressed genes after upregulation of circ_0098181. **(C)** The protein interaction diagram illustrated that circ_0098181 might have the promising potential to affect the inflammatory pathway. **(D, E)** GSEA analysis in circ_0098181 vs. NC. **(F)** KEGG enrichment analysis of the inflammation-related genes. **(G)** Downregulation of TNF-α, Fas, Bcl3, and Cxcl11 mRNA level after circ_0098181 treatment. **(H)** MPO staining to observe neutrophil inflammation infiltration in the four groups (n = 5).

We identified differentially expressed genes between HSC-T6 cells treated with circ_0098181 plasmids (n = 3) and control plasmids (n = 3). Among these differential genes, 255 genes showed a downward trend, while 116 genes were significantly upregulated compared to the NC group (Figures 5A, B). Heatmap and PPI analysis revealed a marked reduction in several pro-inflammatory cytokines, including TNF-α, Fas, Bcl3, and Cxcl11, following circ_0098181 overexpression in HSC-T6 cells (Figure 5C). The decreased levels of TNF-α, Fas, Bcl3, and Cxcl11 were further confirmed by qRT-PCR (Figure 5G). GSEA enrichment analysis revealed that circ_0098181-regulated genes were primarily enriched in pathways related to inflammatory response and leukocyte activation (Figures 5D, E). KEGG enrichment analysis was also conducted, and results highlighted the involvement of inflammation-related genes after circ_0098181 treatment (Figure 5F; Supplementary Figure S4). Additionally, MPO staining was performed to assess neutrophil infiltration across the four groups. Compared to the CCl_4_+Ad-NC group, MPO level of CCl_4_+Ad-circ_0098181 group was significantly reduced, indicating decreased neutrophil infiltration (Figure 5H).

### 3.6 Circ_0098181 binds PKM2 to regulate its nuclear translocation

**FIGURE 6:**
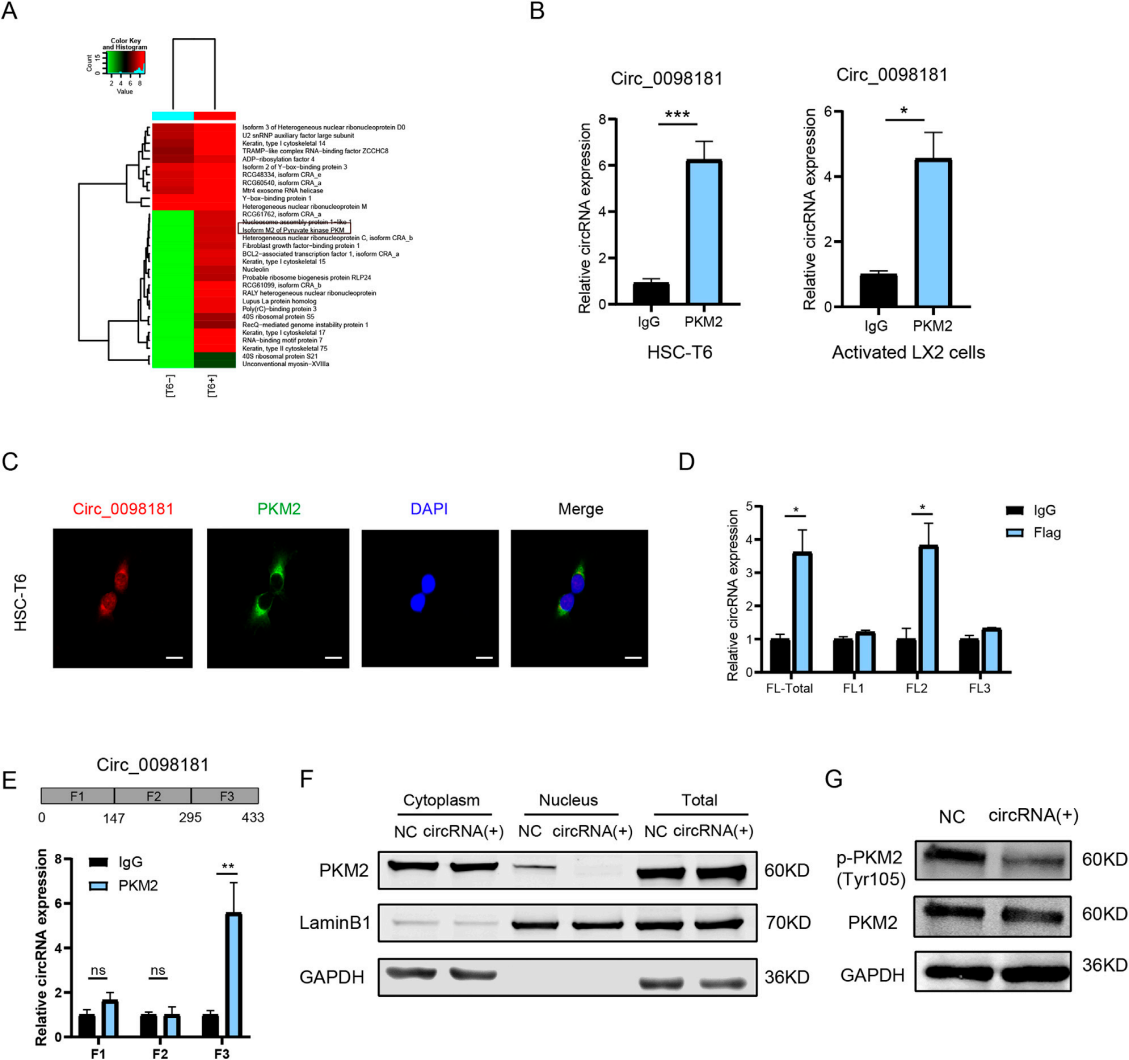
Circ_0098181 binds PKM2 to regulate its nuclear translocation. **(A)** RNA pull-down and mass spectrum results elucidated the potential direct RNA binding proteins (RBPs) for circ_0098181. **(B)** RIP assays proved the direct combination of PKM2 and circ_0098181 in HSC-T6 and LX2 cells. **(C)** The co-localization of circ_0098181 and PKM2 in HSC-T6. Scale bars, 10 μm. **(D)** The sectional PKM2 plasmids were transfected into HSC-T6 cells, and results confirmed that circ_0098181 combined with the second section of PKM2. **(E)** The segmental circ_0098181 primers were performed RIP experiments, suggesting that PKM2 might bind with the third segment sequence of circ_0098181. **(F)** The nuclear and cytoplasmic separational proteins vindicated that circ_0098181 did not affect the overall protein level, but significantly suppressed the nucleus PKM2. **(G)** The phosphorylation of PKM2 level was also constricted by circ_0098181 overexpression. *p < 0.05, **p < 0.01, ***p < 0.001.

In our previous study, hsa_circ_0098181 sponged miR-18a-3p to regulate PPARA in HCC (Luo et al., 2022), but this axis was ineffective in HSCs (Supplementary Figure S5A). To investigate alternative mechanisms of circ_0098181 in fibrosis, RNA pull-down and mass spectrometry were conducted in HSC-T6 cells to identify the direct binding proteins of this circRNA. The potential interacting proteins are presented in the heatmap (Figure 6A). Among the identified target proteins, we focused on PKM2, a key regulator of inflammation and fibrosis (Rao et al., 2022), and YBX1, a well-known RNA-binding protein (Xu J. et al., 2020). Further RIP experiments vindicated that circ_0098181 was able to interact with PKM2, but not with YBX1 (Figure 6B, Supplementary Figure S5B). Given that the interaction between circRNAs and proteins is location-dependent, co-localization of PKM2 and circ_0098181 was assessed using fluorescence staining. As expected, PKM2 and circ_0098181 showed strong co-localization in HSC-T6, LX2, and primary rat HSCs (Figure 6C, Supplementary Figure S6A). To further identify the specific binding regions of circ_0098181, the PKM2 sequence was divided into three equal segments, and RIP assays located the binding site within the second segment of PKM2 (Figure 6D). Additionally, the 443 bp sequence of circ_0098181 was split into three fragments, with RIP assays indicating that PKM2 binds to the third segment of circ_0098181 (Figure 6E). Given that the biological function of PKM2 depends on its localization and form (Wong et al., 2015), we examined PKM2 protein levels in the whole cell, nucleus, and cytoplasm after circ_0098181 overexpression in HSC-T6 cells. The results showed a significant reduction of PKM2 in the nucleus of circ_0098181-treated HSC-T6 cells, with no notable changes in overall cellular or cytoplasmic PKM2 levels (Figure 6F). Additionally, the level of phosphorylated PKM2 (p-PKM2, Tyr105) was significantly decreased following circ_0098181 treatment (Figure 6G).

## 4 Discussion

Constant damage of liver parenchymal cells, continuous hepatic inflammation, and persistent accumulation of extracellular matrix are the basic pathology of liver cirrhosis (Roehlen et al., 2020). Fibrogenesis, driven by HSCs activation, is the central pathologic signature in the natural progress from liver disease to cirrhosis (Zhang et al., 2016). HSCs activation, identified as HSCs transferring from quiescent cells into fibrogenic myofibroblasts, has been considered as the crucial trigger of fibrogenesis in recent years (Tsuchida and Friedman, 2017). Therefore, inhibiting the activation of HSCs remains the most promising therapeutic approach for the management of liver fibrosis and cirrhosis. In this study, we demonstrated the inhibitory effects of circ_0098181, a circRNA significantly downregulated in cirrhotic tissues, on the activation, proliferation, and migration of HSCs, and the development of liver fibrosis. Furthermore, we elucidated the ADAR1-dependent mechanism underlying circ_0098181 cyclization and identified PKM2 as the direct target mediating its biological function.

CircRNAs, with strong stability due to the highly conservative ring structures, have relatively long half-lives and are resistant to RNase R, thus, becoming a research hotspot nowadays (Patop et al., 2019). Previous studies have aimed to identify circRNAs with significantly altered expression in cirrhosis (Zeng et al., 2021; Wang et al., 2023). Several circRNAs, including circCREBBP, circFBXW4, circPSD3, circ_0070963, and circ_0004018, have been reported to be differentially expressed during HSC activation and liver fibrosis (Wang et al., 2023). In our study, we identified a fibrosis-specific profile consisting of 26 overexpressed and 41 downregulated circRNAs in liver fibrotic tissues. Among them, circ_0098181 emerged as a key circRNA, previously studied in HCC (Luo et al., 2022), showing a significant reduction in both activated HSCs and cirrhotic tissues. Given the progressive decline of circ_0098181 levels from normal to cirrhotic tissues and ultimately to HCC, we hypothesize that circ_0098181 might play a crucial role in liver fibrosis and carcinogenesis.

Emerging evidence suggests that circRNAs have potential as diagnostic biomarkers and therapeutic targets, with several studies exploring their role in chronic liver disease (Chien et al., 2020; Meng et al., 2022). For example, circ-SCAR, located in the mitochondria of fibroblasts, has been shown to improve nonalcoholic steatohepatitis (NASH) by inhibiting mitochondrial ROS (mROS) output (Zhao et al., 2020). Additionally, circ-MTO1, derived from the MTO1 gene, has been found to suppress liver fibrosis by regulating the miR-17-5p/Smad7 axis (Wang et al., 2019). Some circRNAs have also been implicated in drug resistance. For instance, circRNA-SORE has been shown to mediate sorafenib resistance in HCC by spreading resistance through exosomes (Xu J. et al., 2020). Silencing this highly expressed circRNA in sorafenib-resistant HCC cells enhanced the cell-killing effect and overcame drug resistance. In our study, exogenous administration of circ_0098181 significantly suppressed the proliferation, migration, and collagen production of different HSCs and inhibited fibrogenesis in a CCl_4_-induced fibrosis model. Moreover, overexpressing circ_0098181 with adenovirus decreased the ALT level. Given that adenovirus could both infected the HSCs and hepatocytes, we believed that this reduction might due to the upregulation of circ_0098181 in hepatocytes. These findings demonstrate the anti-fibrotic effects of circ_0098181 both *in vitro* and *in vivo*, highlighting its potential as a therapeutic target for the treatment of liver fibrosis and cirrhosis.

It has been suggested that circRNA biogenesis should be a focus in circRNA research (Nielsen et al., 2022). According to previous studies (Kristensen et al., 2019), circular RNAs are typically generated through three mechanisms: intron reverse complementary sequence-driven cyclization, RNA-binding protein-driven cyclization, and lasso-driven cyclization. Based on our data from circBase and sequence alignment analysis, circ_0098181 is derived from the SOX5 gene, with its flanking introns enriched in reverse complementary sequences, but lacking paired Alu sequences. Thus, we inferred that circ_0098181 was generated by RNA binding protein-driven cyclization. We then examined the role of two well-researched RNA-binding proteins, ADAR1 and QKI, in the regulation of circ_0098181 biogenesis. Both ADAR1 and QKI are known to competitively bind to ICSs in the flanking introns of circRNAs, either enhancing or inhibiting circRNA synthesis (Ivanov et al., 2015). Previous studies have shown that high ADAR1 expression is associated with liver cirrhosis development and poor prognosis in HCC (Chan et al., 2014). In our study, only ADAR1 silencing increased circ_0098181 expression, whereas QKI knockdown had no effect. Further partial deletion experiments demonstrated the essential role of ICSs in both flanking regions. Thus, ADAR1 is the key RNA-binding protein involved in regulating circ_0098181 biogenesis.

To investigate the potential mechanism, we performed RNA sequencing to identify downstream pathways of circ_0098181 in fibrogenesis. The results revealed a decrease in several inflammation-related genes, including TNF-α, Fas, Cxcl11, Bcl3, etc., following circ_0098181 overexpression. GSEA and KEGG pathway analysis further indicated that cytokine-cytokine receptor interactions and TNF signaling were among the most enriched pathways. Since chronic inflammation is known to contribute to the progression of fibrosis and cirrhosis, these findings suggest that circ_0098181 may alleviate inflammation, thereby inhibiting fibrogenesis.

PKM2, a key enzyme in glycolysis, has been shown to contribute to Toll-like receptor (TLR) and NF-κB-mediated inflammation (Zhang et al., 2021; Gu et al., 2021). Additionally, PKM2 plays a role in regulating hepatic macrophages in the progression of NASH and HCC (Xu F. et al., 2020; Hou et al., 2020). Our RNA pull-down and mass spectrometry analysis revealed an interaction between circ_0098181 and PKM2. Given the critical role of PKM2 in inflammation and liver fibrosis, we hypothesized that PKM2 might be a direct target of circ_0098181. This was further confirmed by RIP assays, which demonstrated a binding interaction between circ_0098181 and PKM2. PKM2 is known to exist in three forms—monomer, dimer, and tetramer—with different functions. The dimer typically resides in the nucleus, where it acts as a protein kinase, while the tetramer is abundant in the cytoplasm, showing high pyruvate kinase activity and promoting glucose metabolism (Zhang et al., 2019; Liu et al., 2021). In the liver, PKM2 is predominantly expressed in activated HSCs, rather than in quiescent HSCs or hepatocytes, and PKM2 dimer in the nucleus induced HSCs activation (Zheng et al., 2020). We sought to investigate circ_0098181's influence on PKM2 forms and translocation. Our results verified that circ_0098181 significantly reduced nuclear PKM2 protein levels. As reported, phosphorylated PKM2 (p-PKM2) reflects the nuclear form of PKM2 (Kalaiarasan et al., 2014). Our Western blot analysis confirmed that circ_0098181 reduced the nuclear translocation of PKM2. Additionally, as reported (Fei et al., 2024), dimeric PKM2 can act as a coactivator of β-catenin and c-Myc, which are key transcription factors in fibrogenesis (Duspara et al., 2021; Liu et al., 2015), thereby leading to the promotion of liver fibrosis.

In conclusion, circ_0098181, which is significantly downregulated in activated HSCs and cirrhotic liver tissues, demonstrates a strong anti-fibrotic effect by binding to PKM2, repressing its nuclear translocation, and inhibiting hepatic inflammation. These findings offer proof-of-concept for a potential therapeutic strategy targeting circ_0098181 in the treatment of liver fibrosis.

## Data Availability

The original contributions presented in the study are included in the article/Supplementary Material, further inquiries can be directed to the corresponding authors.
