# Supplementary figures and images for: Circ_0098181 binds PKM2 to attenuate liver fibrosis
Source: Front Pharmacol. 2025 Apr 3;16:1517250. doi: 10.3389/fphar.2025.1517250 (PMC12003362; doi:10.3389/fphar.2025.1517250)

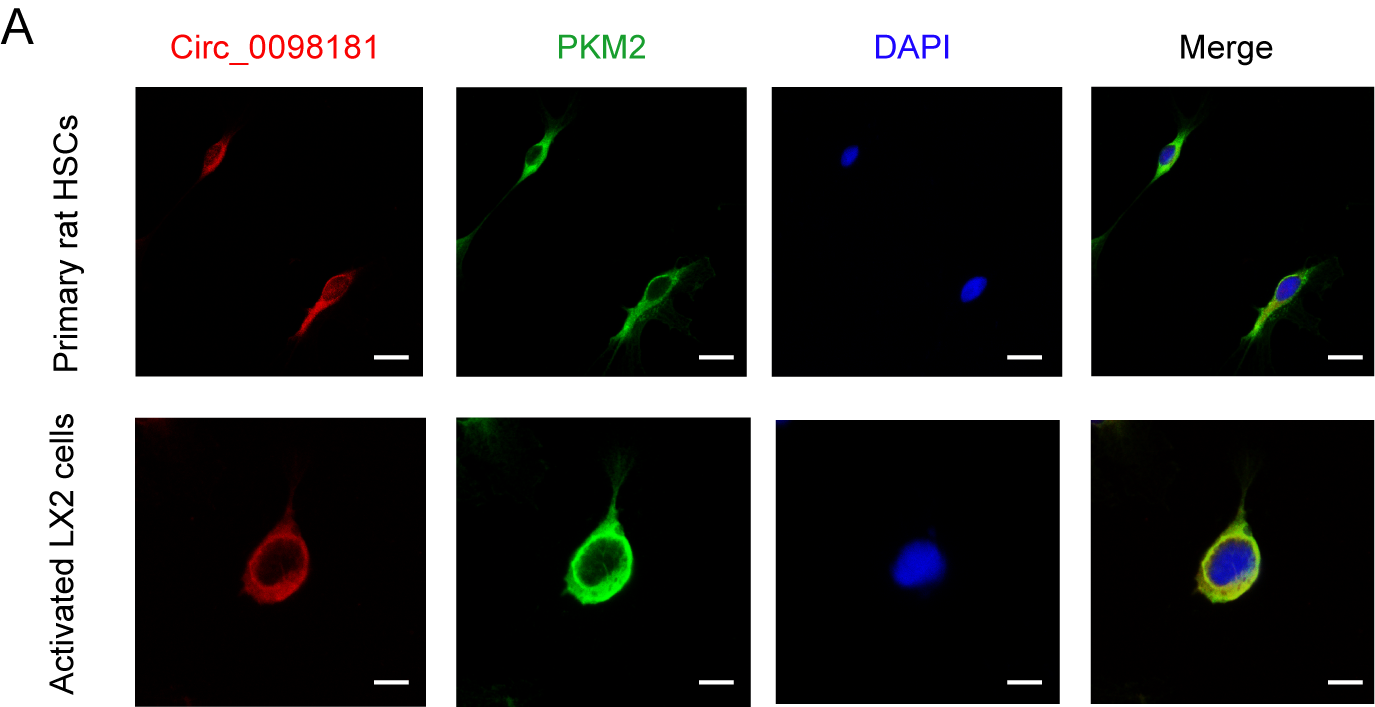

Supplement: Supplementary file 1 [file Image6.tif]

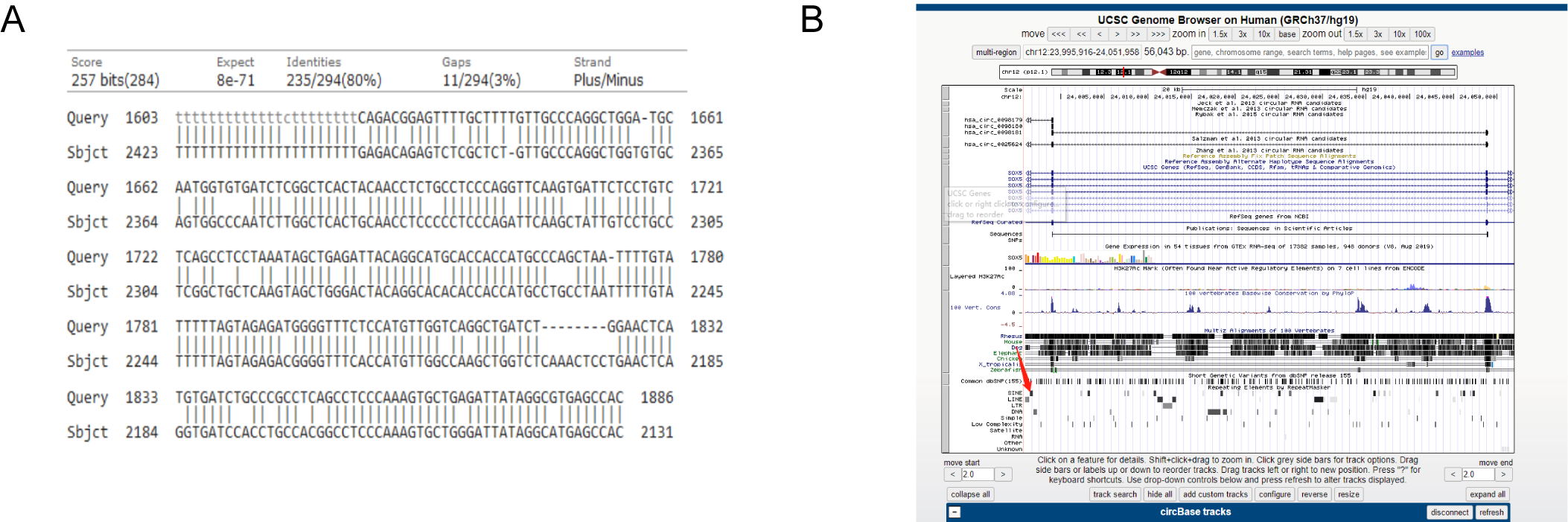

Supplement: Supplementary file 3 [file Image3.tif]

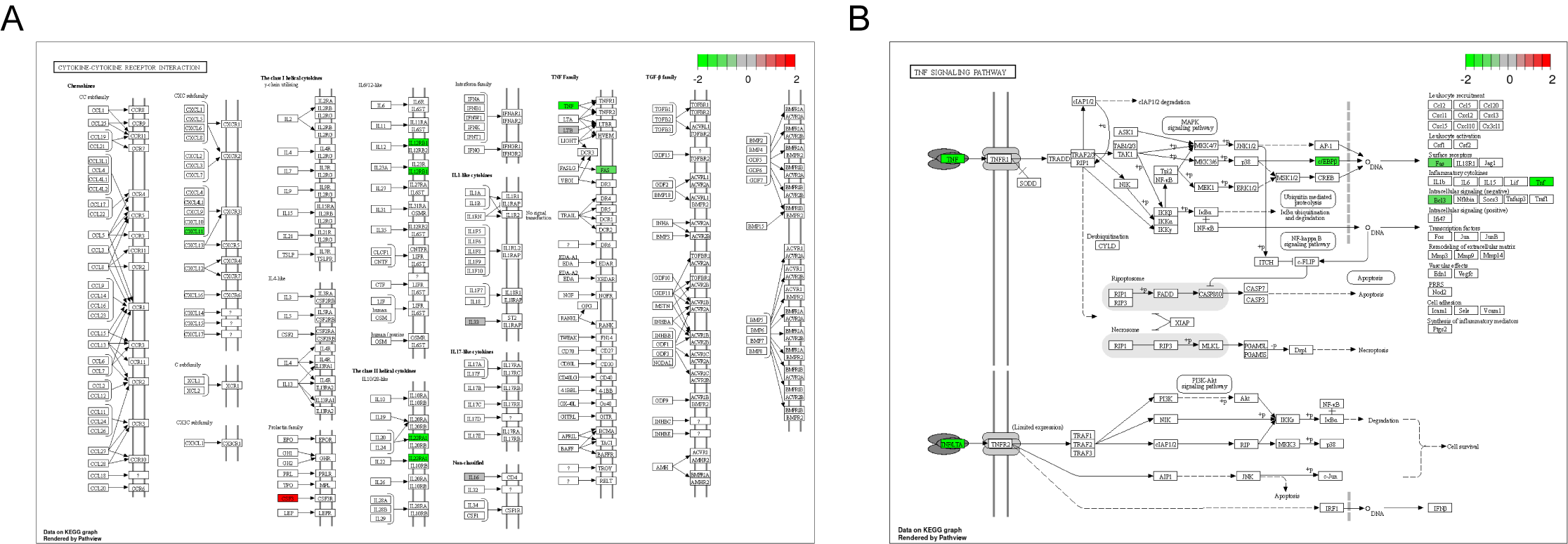

Supplement: Supplementary file 4 [file Image4.tif]

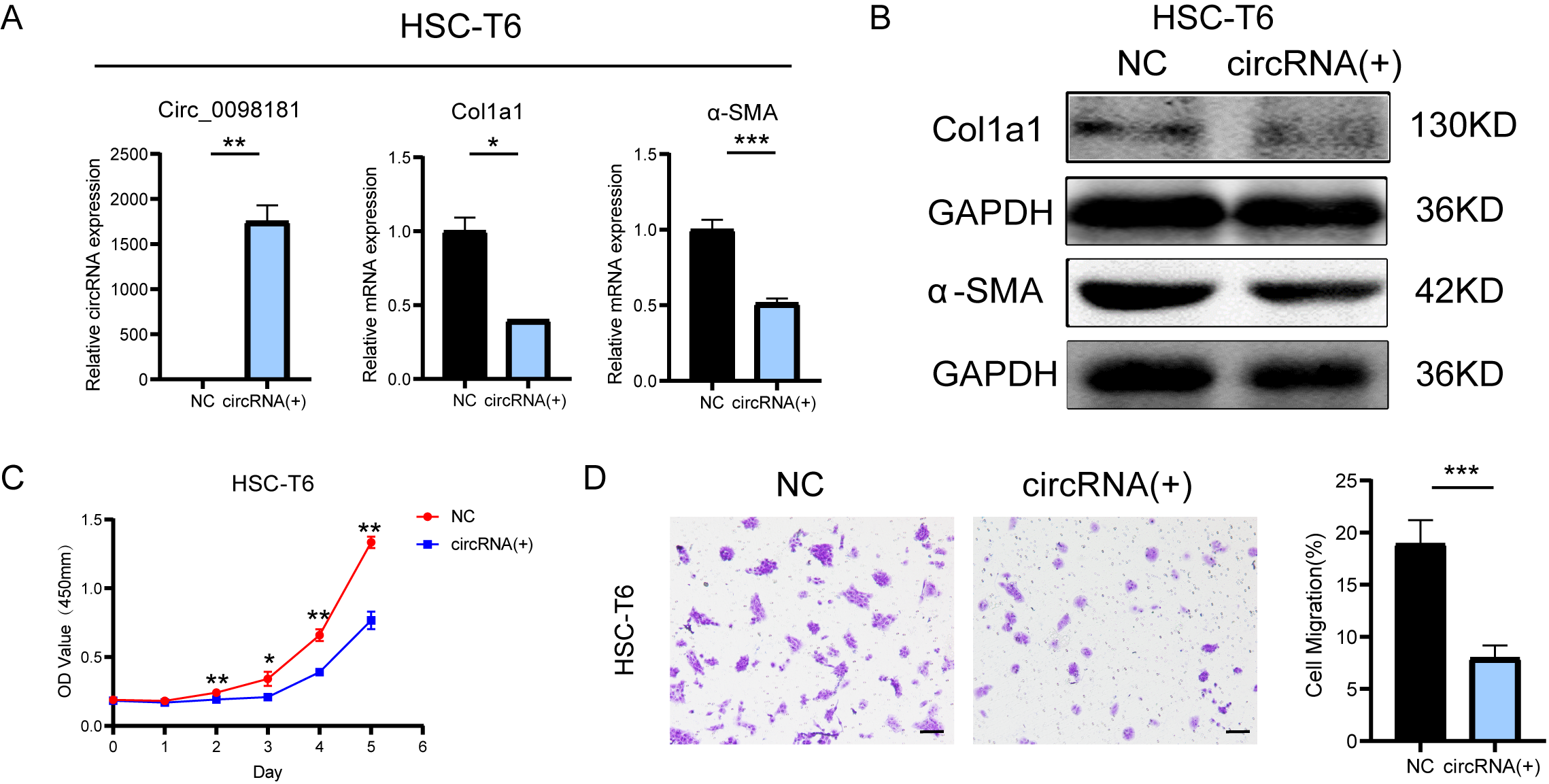

Supplement: Supplementary file 5 [file Image2.tif]

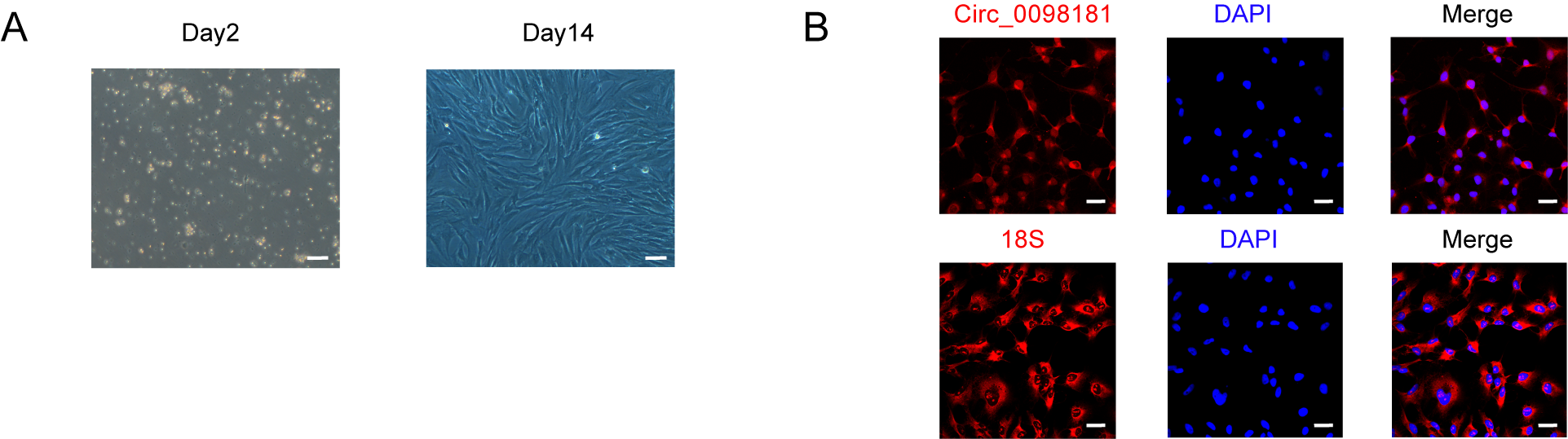

Supplement: Supplementary file 6 [file Image1.tif]

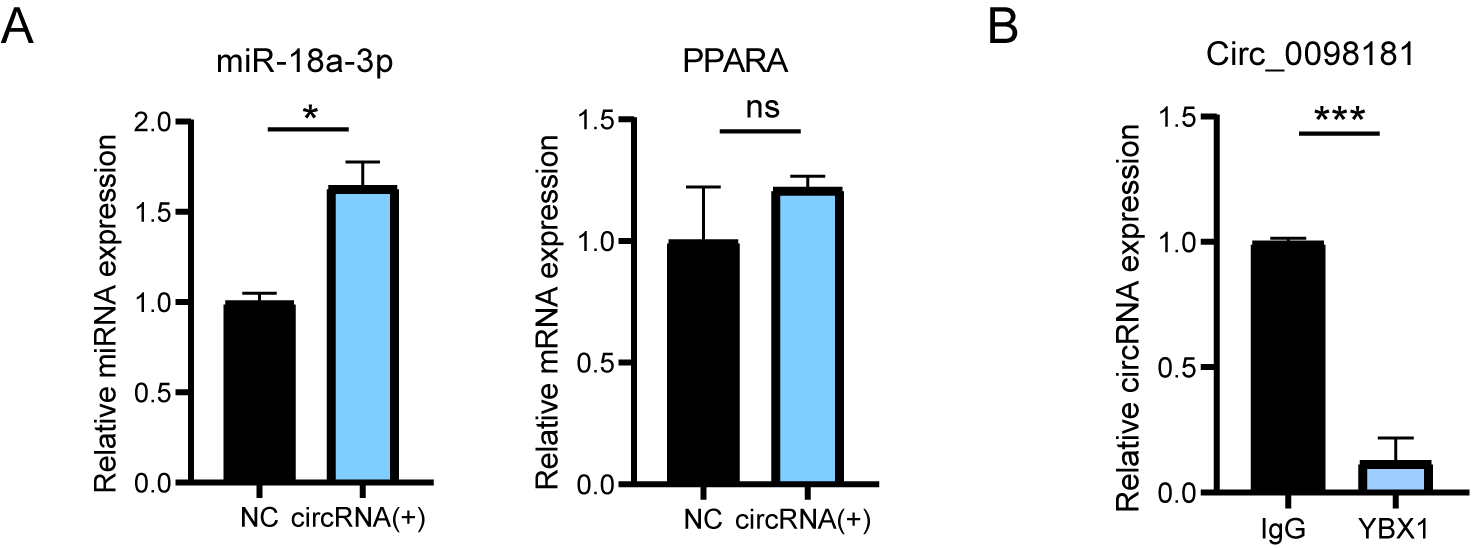

Supplement: Supplementary file 8 [file Image5.tif]
