# Supplementary material for: Circ_0098181 binds PKM2 to attenuate liver fibrosis
Source: Front Pharmacol. 2025 Apr 3;16:1517250. doi: 10.3389/fphar.2025.1517250 (PMC12003362; doi:10.3389/fphar.2025.1517250)
Supplement: Supplementary file 2 [file Table1.docx]

**Table S1** **Demographic characteristics of the participants**

|  | Control (*n*=5) | Liver cirrhosis(*n*=5) | Hepatocellular carcinoma (n=5) | *p* value |  |
| --- | --- | --- | --- | --- | --- |
| Demographics |  |  |  |  |  |
| Gender, female(%) | 4 (80%) | 3 (60%) | 1 (20%) | 0.153 |  |
| Primary etiology-no.(%) |  |  |  |  |  |
| HBV |  | 4 (80%) | 5 (100%) |  |  |
| Undefined |  | 1 (20%) |  |  |  |
| MELD score | 9.41±3.46 | 15.20±5.49 | 9.80±3.49 | 0.094 |  |
| Laboratory characteristics |  |  |  |  |  |
| TBIL -μmol/L | 13.72±6.71 | 63.96±72.53 | 11.66±5.76 | 0.127 |  |
| DBIL -μmol/L | 4.44±1.88 | 42.28±54.13 | 5.28±2.45 | 0.134 |  |
| ALB -g/L | 58.02±11.96 | 37.26±8.48 | 43.32±2.81 | 0.007 |  |
| ALT -U/L | | 39.40±50.67 | 35.60±11.84 | 48.80±20.83 | 0.805 |
| Creatinine -μmol/L | 66.00±9.67 | 63.60±14.72 | 74.20±7.50 | 0.317 |  |
| Prothrombin time-sec | 12.10±1.36 | 14.90±4.20 | 11.80±1.00 | 0.160 |  |
| INR | 1.02±0.11 | 1.30±0.39 | 1.00±0.11 | 0.142 |  |
| WBC -10^9/L | 4.32±0.80 | 3.35±0.73 | 5.76±1.50 | 0.016 |  |
| HB -g/L | 124.75±12.04 | 101.80±31.04 | 149.40±19.92 | 0.024 |  |
| PLT -10^9/L | 239.75±47.47 | 107.00±60.97 | 205.60±72.00 | 0.020 |  |

*MELD* Model for End-Stage Liver Disease, *TBIL* total bilirubin, *DBIL* direct bilirubin, *ALB* albumin, *ALT* alanine aminotransferase, *INR* international normalized ratio, *WBC* white blood cell, *HB* hemoglobin, *PLT* platelet

# Table S2 Primers for RT-PCR

| Gene | Forward sequence | Reverse sequence |
| --- | --- | --- |
| homo-GAPDH  has_circ_0098181  homo-α-SMA  homo-Col1a1  rno-GAPDH  rno- circ_0098181  rno-α-SMA | TCTCTGCTCCTCCTGTTC  TGTTGACACCTTGAAGCAGAG  AAAAGACAGCTACGTGGGTGA  TTCCCGGTGAATTCGGTCTC  TGGAGTCTACTGGCGTCTT  TCAGAAGGCGGAAGAAGGTG  CCGAGATCTCACCGACTACC | GTTGACTCCGACCTTCAC  CATCTGCTTCCCCATACGGA  GCCATGTTCTATCGGGTACTTC  ACCTCGGATTCCAATAGGACCAG  TGTCATATTTCTCGTGGTTCA  AAGACATCCTTCCGGCTCGT  TCCAGAGCGACATAGCACAG |
| rno- Col1a1  rno- ADAR  rno-QKI  ICS1  ICS3  segmented-F1  segmented-F2  segmented-F3  rno-TNF-α  rno-Fas  rno-Cxcl11  rno-Bcl3 | GCTCGTGGATTGCCTGGAACAG  CCACAGATGCTTCAACGCTCT  ATGTGCCTGAAAAGAATACCCT  TTCTCCTGTCTCAGCCTCCT  CGGTGGCTCATGCCTATAAT  ATGGAGAGGTAGCCATGGTG  TCTCTGCTGACGCAAGAGAC  ACAGAGTGGCGAGTCCTTGT  ATGGGCTCCCTCTCATCAGT  AGCTGCTCCAGTGCTGGTAT  TATGATCATCTGGGCCACAA  GCTGAACCTGCCTACTCACC | CACCGACAGCACCATCGTTACC  CCTTTCACACAGCGATTCCCA  GCCTCGGACCATTATTTTACATCC  GGGCAGATCACATGAGTTCCA  CACCATGCCTGCCTAATTTT  GAGTGAGGCTTGTTGGGAAA  ATGAGTTGTGTGGGGCAAAT  TTCCATTTTCCTCTGCTTCAA  GGCTGGGTAGAGAACGGATG  CATAGGTGGCAGGCTCTCTC  CTTGATTGCTGCCATTTTGA  GGCCGTCTTGTTATTCTGGA |

# Table S3 Antibodies

| Antibodies | Company | Dilution |
| --- | --- | --- |
| α-SMA  Col1a1  GAPDH  ADAR1  PKM2  YBX1  p-PKM2  LaminB1  Flag | Bioss, bs-10196R  Bioss, bs-10423R  ABclonal, A19056  Proteintech, 14330-1-AP  CST, 4053S  Abcam, ab76149  Immunoway, YP1444  Abcam, ab133741  Abcam, ab205606 | 1:1000  1:1000  1:10000  1:50  1:1000  1:50  1:1000  1:1000  1:50 |

# Table S4 SiRNA sequence

| Name | Sequence |
| --- | --- |
| ADAR-rat-siRNA-1- 2694  ADAR-rat-siRNA-2-1489  ADAR-rat-siRNA-3- 3469  Negative siRNA  QKI-rat-siRNA-1-913  QKI-rat-siRNA-2-785  QKI-rat-siRNA-3-608 | GAGAGAAGCUCCAGAUAAA  CAUCAAUGGUCGAGAGUUU  GGACAUGGGCUAUGGGAAU  UUCUCCGAACGUGUCACGU  GAGCAGAAAUCAAGCUGAA  AAUAAUGGUCCGAGGCAAA  AAAUGGCAGUACAGAGAAA |

# Table S5 Plasmids for flanking sequence

| Name | Sequence |
| --- | --- |
| P1(whole cric_0098181 sequence, ICS1, and ICS3) | ATATGTAGTTAGTGTAAGTCATATAATGTAAGCTATATAAATATATGCACACTTGTGGTGCCTTTTTTTTTTTTTCTTTTTTTTCAGACGGAGTTTTGCTTTTGTTGCCCAGGCTGGATGCAATGGTGTGATCTCGGCTCACTACAACCTCTGCCTCCCAGGTTCAAGTGATTCTCCTGTCTCAGCCTCCTAAATAGCTGAGATTACAGGCATGCACCACCATGCCCAGCTAATTTTGTATTTTTAGTAGAGATGGGGTTTCTCCATGTTGGTCAGGCTGATCTGGAACTCATGTGATCTGCCCGCCTCAGCCTCCCAAAGTGCTGAGATTATAGGCGTGAGCCACTGCAACCGGCCTGTGGTGCCTTCTTAATGAGACTTTTTTCTGCCCAAGTGTTTCAACACTTTGATAGATGGACAAAAATTTAAGAATCTAATGTTATTGGTGATTTTGTTGTCAATATTTTTGAGAAGTACTATGGGTCATAGTAAACATGAATAATACAACCAACAGATTGATATATCTATATGTAATTTGTTATATAGTTTACTTTTCTGACTTAGTAGCCTATTATTGAAGTATAACCGTGGTTGCTTTTAGATGTCTTCCAAGCGACCAGCCTCTCCGTATGGGGAAGCAGATGGAGAGGTAGCCATGGTGACAAGCAGACAGAAAGTGGAAGAAGAGGAGAGTGACGGGCTCCCAGCCTTTCACCTTCCCTTGCATGTGAGTTTTCCCAACAAGCCTCACTCTGAGGAATTTCAGCCAGTTTCTCTGCTGACGCAAGAGACTTGTGGCCATAGGACTCCCACTTCTCAGCACAATACAATGGAAGTTGATGGCAATAAAGTTATGTCTTCATTTGCCCCACACAACTCATCTACCTCACCTCAGAAGGCAGAAGAAGGTGGGCGACAGAGTGGCGAGTCCTTGTCTAGTACAGCCCTGGGAACTCCTGAACGGCGCAAGGGCAGTTTAGCTGATGTTGTTGACACCTTGAAGCAGAGGAAAATGGAAGAGCTCATCAAAAACGAGCCGGAAGGAATTAAGTGCATTTTTTACTTAATGATATTTTCAAGTTGCAAAGGGCTTAAGGACATAACCCTATCATAAGTCTAGGAGCATCTGTACTTTTAAAATATTGTGCTTCCTATTTCATTTAGAATTATAGTATGAGTGTGTCTTCTGCCAAGTAATATACTTTGAAAAGAAATAACTGCCAAGGCTGGGCACGGTGGCTCATGCCTATAATCCCAGCACTTTGGGAGGCCGTGGCAGGTGGATCACCTGAGTTCAGGAGTTTGAGACCAGCTTGGCCAACATGGTGAAACCCCGTCTCTACTAAAAATACAAAAATTAGGCAGGCATGGTGGTGTGTGCCTGTAGTCCCAGCTACTTGAGCAGCCGAGGCAGGACAATAGCTTGAATCTGGGAGGGGGAGGTTGCAGTGAGCCAAGATTGGGCCACTGCACACCAGCCTGGGCAACAGAGCGAGACTCTGTCTCAAAAAAAAAAAAAAAAAAAAAAAAAAAGGAGAAATGACTGTCAAATATTCTGAAGTATGGCCATGATTTGCTATTGTAATTTATACATTCACTTTATTTTTTTTAAAAACAGTTTTAGATTTACGGAAAAATTAAGC |
| P2(including ICS1 and full length of circ_0098181) | ATATGTAGTTAGTGTAAGTCATATAATGTAAGCTATATAAATATATGCACACTTGTGGTGCCTTTTTTTTTTTTTCTTTTTTTTCAGACGGAGTTTTGCTTTTGTTGCCCAGGCTGGATGCAATGGTGTGATCTCGGCTCACTACAACCTCTGCCTCCCAGGTTCAAGTGATTCTCCTGTCTCAGCCTCCTAAATAGCTGAGATTACAGGCATGCACCACCATGCCCAGCTAATTTTGTATTTTTAGTAGAGATGGGGTTTCTCCATGTTGGTCAGGCTGATCTGGAACTCATGTGATCTGCCCGCCTCAGCCTCCCAAAGTGCTGAGATTATAGGCGTGAGCCACTGCAACCGGCCTGTGGTGCCTTCTTAATGAGACTTTTTTCTGCCCAAGTGTTTCAACACTTTGATAGATGGACAAAAATTTAAGAATCTAATGTTATTGGTGATTTTGTTGTCAATATTTTTGAGAAGTACTATGGGTCATAGTAAACATGAATAATACAACCAACAGATTGATATATCTATATGTAATTTGTTATATAGTTTACTTTTCTGACTTAGTAGCCTATTATTGAAGTATAACCGTGGTTGCTTTTAGATGTCTTCCAAGCGACCAGCCTCTCCGTATGGGGAAGCAGATGGAGAGGTAGCCATGGTGACAAGCAGACAGAAAGTGGAAGAAGAGGAGAGTGACGGGCTCCCAGCCTTTCACCTTCCCTTGCATGTGAGTTTTCCCAACAAGCCTCACTCTGAGGAATTTCAGCCAGTTTCTCTGCTGACGCAAGAGACTTGTGGCCATAGGACTCCCACTTCTCAGCACAATACAATGGAAGTTGATGGCAATAAAGTTATGTCTTCATTTGCCCCACACAACTCATCTACCTCACCTCAGAAGGCAGAAGAAGGTGGGCGACAGAGTGGCGAGTCCTTGTCTAGTACAGCCCTGGGAACTCCTGAACGGCGCAAGGGCAGTTTAGCTGATGTTGTTGACACCTTGAAGCAGAGGAAAATGGAAGAGCTCATCAAAAACGAGCCGGAAG |
| P3(including ICS3 and full length of circ_0098181) | GATGTCTTCCAAGCGACCAGCCTCTCCGTATGGGGAAGCAGATGGAGAGGTAGCCATGGTGACAAGCAGACAGAAAGTGGAAGAAGAGGAGAGTGACGGGCTCCCAGCCTTTCACCTTCCCTTGCATGTGAGTTTTCCCAACAAGCCTCACTCTGAGGAATTTCAGCCAGTTTCTCTGCTGACGCAAGAGACTTGTGGCCATAGGACTCCCACTTCTCAGCACAATACAATGGAAGTTGATGGCAATAAAGTTATGTCTTCATTTGCCCCACACAACTCATCTACCTCACCTCAGAAGGCAGAAGAAGGTGGGCGACAGAGTGGCGAGTCCTTGTCTAGTACAGCCCTGGGAACTCCTGAACGGCGCAAGGGCAGTTTAGCTGATGTTGTTGACACCTTGAAGCAGAGGAAAATGGAAGAGCTCATCAAAAACGAGCCGGAAGGAATTAAGTGCATTTTTTACTTAATGATATTTTCAAGTTGCAAAGGGCTTAAGGACATAACCCTATCATAAGTCTAGGAGCATCTGTACTTTTAAAATATTGTGCTTCCTATTTCATTTAGAATTATAGTATGAGTGTGTCTTCTGCCAAGTAATATACTTTGAAAAGAAATAACTGCCAAGGCTGGGCACGGTGGCTCATGCCTATAATCCCAGCACTTTGGGAGGCCGTGGCAGGTGGATCACCTGAGTTCAGGAGTTTGAGACCAGCTTGGCCAACATGGTGAAACCCCGTCTCTACTAAAAATACAAAAATTAGGCAGGCATGGTGGTGTGTGCCTGTAGTCCCAGCTACTTGAGCAGCCGAGGCAGGACAATAGCTTGAATCTGGGAGGGGGAGGTTGCAGTGAGCCAAGATTGGGCCACTGCACACCAGCCTGGGCAACAGAGCGAGACTCTGTCTCAAAAAAAAAAAAAAAAAAAAAAAAAAAGGAGAAATGACTGTCAAATATTCTGAAGTATGGCCATGATTTGCTATTGTAATTTATACATTCACTTTATTTTTTTTAAAAACAGTTTTAGATTTACGGAAAAATTAAGC |
| P4(including full length of circ_0098181) | GATGTCTTCCAAGCGACCAGCCTCTCCGTATGGGGAAGCAGATGGAGAGGTAGCCATGGTGACAAGCAGACAGAAAGTGGAAGAAGAGGAGAGTGACGGGCTCCCAGCCTTTCACCTTCCCTTGCATGTGAGTTTTCCCAACAAGCCTCACTCTGAGGAATTTCAGCCAGTTTCTCTGCTGACGCAAGAGACTTGTGGCCATAGGACTCCCACTTCTCAGCACAATACAATGGAAGTTGATGGCAATAAAGTTATGTCTTCATTTGCCCCACACAACTCATCTACCTCACCTCAGAAGGCAGAAGAAGGTGGGCGACAGAGTGGCGAGTCCTTGTCTAGTACAGCCCTGGGAACTCCTGAACGGCGCAAGGGCAGTTTAGCTGATGTTGTTGACACCTTGAAGCAGAGGAAAATGGAAGAGCTCATCAAAAACGAGCCGGAAG |
| P5(NC) | pCDNA3.1 empty plasmid |

# Table S6 Plasmids for segmental PKM2 sequence

| Name | | Sequence |
| --- | --- | --- |
| Flag-total | ATGGATTACAAGGATGACGACGATAAGGCCACCATGCAGTGGAGCTCAGAGAGAGGAGAACGGCTCCTCACGCCTGGGGCCTGCTCTTCAGAAGTCCCCAGCGCCGTTCCTTCCAGATCAGGCGGCTCTCCAGGGCACACCGTATTCAGCTCTGAGCGGTCTTTGCTAGTGAGGCCAAGGAGCCACCCTGAGCCAAAAGGGGAGCATTATGTCACCGGAAGCCCAACCCCAGAGAACCAAAGGACCTCAGCAGCCATGTCGAAGCCCCATAGTGAAGCCGGGACTGCCTTCATTCAGACCCAGCAGCTGCACGCAGCCATGGCTGACACATTCCTGGAGCACATGTGCCGCCTGGACATTGATTCACCACCCATCACAGCCCGGAACACTGGCATCATCTGTACCATTGGCCCAGCTTCCCGATCAGTGGAGACGTTGAAGGAGATGATTAAGTCTGGAATGAATGTGGCTCGTCTGAACTTCTCTCATGGAACTCATGAGTACCATGCGGAGACCATCAAGAATGTGCGCACAGCCACGGAAAGCTTTGCTTCTGACCCCATCCTCTACCGGCCCGTTGCTGTGGCTCTAGACACTAAAGGACCTGAGATCCGAACTGGGCTCATCAAGGGCAGCGGCACTGCAGAGGTGGAGCTGAAGAAGGGAGCCACTCTCAAAATCACGCTGGATAACGCCTACATGGAAAAGTGTGACGAGAACATCCTGTGGCTGGACTACAAGAACATCTGCAAGGTGGTGGAAGTGGGCAGCAAGATCTACGTGGATGATGGGCTTATTTCTCTCCAGGTGAAGCAGAAAGGTGCCGACTTCCTGGTGACGGAGGTGGAAAATGGTGGCTCCTTGGGCAGCAAGAAGGGTGTGAACCTTCCTGGGGCTGCTGTGGACTTGCCTGCTGTGTCGGAGAAGGACATCCAGGATCTGAAGTTTGGGGTCGAGCAGGATGTTGATATGGTGTTTGCGTCATTCATCCGCAAGGCATCTGATGTCCATGAAGTTAGGAAGGTCCTGGGAGAGAAGGGAAAGAACATCAAGATTATCAGCAAAATCGAGAATCATGAGGGGGTTCGGAGGTTTGATGAAATCCTGGAGGCCAGTGATGGGATCATGGTGGCTCGTGGTGATCTAGGCATTGAGATTCCTGCAGAGAAGGTCTTCCTTGCTCAGAAGATGATGATTGGACGGTGCAACCGAGCTGGGAAGCCTGTCATCTGTGCTACTCAGATGCTGGAGAGCATGATCAAGAAGCCCCGCCCCACTCGGGCTGAAGGCAGTGATGTGGCCAATGCAGTCCTGGATGGAGCCGACTGCATCATGCTGTCTGGAGAAACAGCCAAAGGGGACTATCCTCTGGAGGCTGTGCGCATGCAGCACCTGATAGCTCGTGAGGCTGAGGCAGCCATGTTCCACCGCAAGCTGTTTGAAGAACTTGTGCGAGCCTCAAGTCACTCCACAGACCTCATGGAAGCCATGGCCATGGGCAGCGTGGAGGCTTCTTATAAGTGTTTAGCAGCAGCTTTGATAGTTCTGACGGAGTCTGGCAGGTCTGCTCACCAGGTGGCCAGATACCGCCCACGTGCCCCCATCATTGCTGTGACCCGGAATCCCCAGACAGCTCGTCAGGCCCACCTGTACCGTGGCATCTTCCCTGTGCTGTGCAAGGACCCAGTCCAGGAGGCCTGGGCTGAGGACGTGGACCTCCGGGTGAACTTTGCCATGAATGTTGGCAAGGCCCGAGGCTTCTTCAAGAAGGGAGATGTGGTCATTGTGCTGACCGGATGGCGCCCTGGCTCCGGCTTCACCAACACCATGCGTGTTGTTCCTGTGCCGTGA | |
| Flag-PKM2-1 | ATGGATTACAAGGATGACGACGATAAGGCCACCATGCAGTGGAGCTCAGAGAGAGGAGAACGGCTCCTCACGCCTGGGGCCTGCTCTTCAGAAGTCCCCAGCGCCGTTCCTTCCAGATCAGGCGGCTCTCCAGGGCACACCGTATTCAGCTCTGAGCGGTCTTTGCTAGTGAGGCCAAGGAGCCACCCTGAGCCAAAAGGGGAGCATTATGTCACCGGAAGCCCAACCCCAGAGAACCAAAGGACCTCAGCAGCCATGTCGAAGCCCCATAGTGAAGCCGGGACTGCCTTCATTCAGACCCAGCAGCTGCACGCAGCCATGGCTGACACATTCCTGGAGCACATGTGCCGCCTGGACATTGATTCACCACCCATCACAGCCCGGAACACTGGCATCATCTGTACCATTGGCCCAGCTTCCCGATCAGTGGAGACGTTGAAGGAGATGATTAAGTCTGGAATGAATGTGGCTCGTCTGAACTTCTCTCATGGAACTCATGAGTACCATGCGGAGACCATCAAGAATGTGCGCACAGCCACGGAAAGCTTTGCTTCTGACCCCATCCTCTACCGGCCCGTTGCTGTGGCTCTAGACACTAAAGGACCTGAGATCCGAACTGGGCTCATCAAGGGCAGCGGCTGA | |
| Flag-PKM2-2 | ATGGATTACAAGGATGACGACGATAAGGCCACCATGACTGCAGAGGTGGAGCTGAAGAAGGGAGCCACTCTCAAAATCACGCTGGATAACGCCTACATGGAAAAGTGTGACGAGAACATCCTGTGGCTGGACTACAAGAACATCTGCAAGGTGGTGGAAGTGGGCAGCAAGATCTACGTGGATGATGGGCTTATTTCTCTCCAGGTGAAGCAGAAAGGTGCCGACTTCCTGGTGACGGAGGTGGAAAATGGTGGCTCCTTGGGCAGCAAGAAGGGTGTGAACCTTCCTGGGGCTGCTGTGGACTTGCCTGCTGTGTCGGAGAAGGACATCCAGGATCTGAAGTTTGGGGTCGAGCAGGATGTTGATATGGTGTTTGCGTCATTCATCCGCAAGGCATCTGATGTCCATGAAGTTAGGAAGGTCCTGGGAGAGAAGGGAAAGAACATCAAGATTATCAGCAAAATCGAGAATCATGAGGGGGTTCGGAGGTTTGATGAAATCCTGGAGGCCAGTGATGGGATCATGGTGGCTCGTGGTGATCTAGGCATTGAGATTCCTGCAGAGAAGGTCTTCCTTGCTCAGAAGATGATGATTGGACGGTGCAACCGAGCTGGGAAGCCTGTCATCTGTGCTACTCAGATGTGA | |
| Flag-PKM2-3 | ATGGATTACAAGGATGACGACGATAAGGCCACCATGCTGGAGAGCATGATCAAGAAGCCCCGCCCCACTCGGGCTGAAGGCAGTGATGTGGCCAATGCAGTCCTGGATGGAGCCGACTGCATCATGCTGTCTGGAGAAACAGCCAAAGGGGACTATCCTCTGGAGGCTGTGCGCATGCAGCACCTGATAGCTCGTGAGGCTGAGGCAGCCATGTTCCACCGCAAGCTGTTTGAAGAACTTGTGCGAGCCTCAAGTCACTCCACAGACCTCATGGAAGCCATGGCCATGGGCAGCGTGGAGGCTTCTTATAAGTGTTTAGCAGCAGCTTTGATAGTTCTGACGGAGTCTGGCAGGTCTGCTCACCAGGTGGCCAGATACCGCCCACGTGCCCCCATCATTGCTGTGACCCGGAATCCCCAGACAGCTCGTCAGGCCCACCTGTACCGTGGCATCTTCCCTGTGCTGTGCAAGGACCCAGTCCAGGAGGCCTGGGCTGAGGACGTGGACCTCCGGGTGAACTTTGCCATGAATGTTGGCAAGGCCCGAGGCTTCTTCAAGAAGGGAGATGTGGTCATTGTGCTGACCGGATGGCGCCCTGGCTCCGGCTTCACCAACACCATGCGTGTTGTTCCTGTGCCGTGA | |
