# Supplementary material for: Circ_0098181 binds PKM2 to attenuate liver fibrosis
Source: Front Pharmacol. 2025 Apr 3;16:1517250. doi: 10.3389/fphar.2025.1517250 (PMC12003362; doi:10.3389/fphar.2025.1517250)
Supplement: Supplementary file 7 [file DataSheet2.docx]

**Supplementary Figure Legends**

**Fig.S1 The activation of primary HSCs on Day14 and FISH location of circ_0098181 in LX2.**

(A) Cell morphology on Day2 and Day 14 of primary HSCs under inverted microscope. Scale bars, 50μm. (B) The location circ_0098181 and the positive cytoplasmic control (18S) with FISH probes. Scale bars, 10μm.

**Fig.S2 Circ_0098181 represses the biological function of HSC-T6.**

(A-B) Upregulating circ_0098181 inhibited the expression of Col1a1 and α-SMA in HSC-T6 cells. (C) The proliferation of HSC-T6 cells was blocked by exogenous circ_0098181 delivery. (D) Increasing circ_0098181 could blunt the ability of HSC-T6 cells’ migration. Scale bars, 50μm. *p < 0.05, **p < 0.01, ***p < 0.001.

**Fig.S3 The flanking sequence of circ_0098181.**

(A) The flanking sequence of circ_0098181 intron was found to have highly reverse complementary sequences (about 80%). (B) Alu sequence was not exist in flanking sequence.

**Fig.S4 The top 2 pathways by KEGG enrichment analysis.**

Cytokine-cytokine receptor interaction (B) TNF signaling pathway. (Red, the up-regulated genes; Blue, down-regulated genes.)

**Fig.S5 circ_0098181 did not have the miR-18a-3p axis, and not combined with YBX1.**

(A) Exogenous circ_0098181 delivery with did not regulate miR-18a-3p/PPARA axis in HSC-T6 cells. (B) Compared with IgG group, YBX1 did not combined with circ_0098181. *p < 0.05, **p < 0.01, ***p < 0.001.

**Fig.S6 Co-localization between PKM2 and circ_0098181 in primary rat HSCs and LX2.**

(A) Cellular immunofluorescence verified the co-localization of circ_0098181 and PKM2 in primary rat HSCs and LX2. Scale bars, 10μm.
