# Supplementary material for: Circ_0098181 binds PKM2 to attenuate liver fibrosis
Source: Front Pharmacol. 2025 Apr 3;16:1517250. doi: 10.3389/fphar.2025.1517250 (PMC12003362; doi:10.3389/fphar.2025.1517250)
Supplement: Supplementary file 9 [file DataSheet1.docx]

1. Full uncropped Gels and Blots images for Figure2C

NC circ+


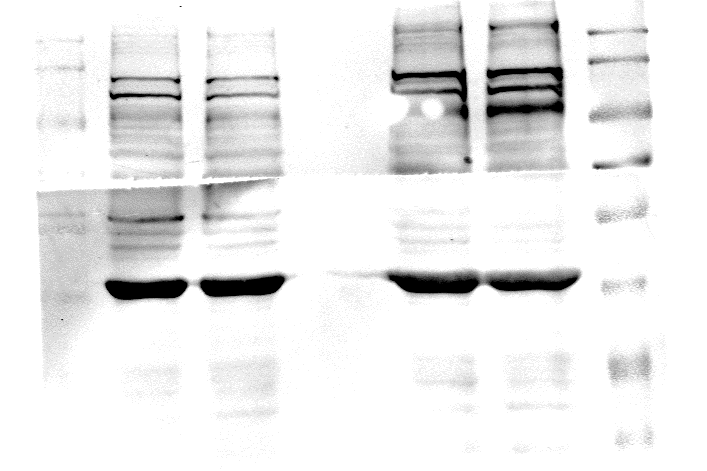


col1a1(130kd)


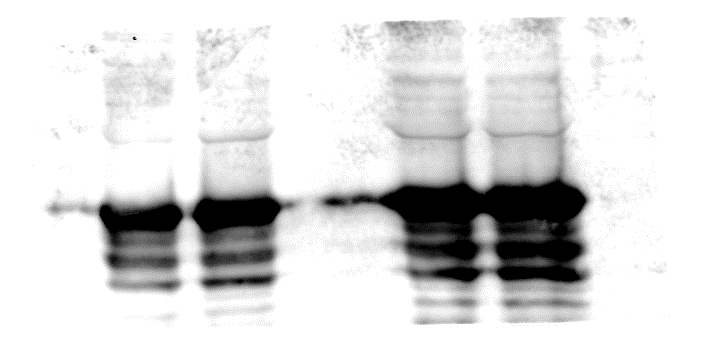


GAPDH

NC circ+ circ+


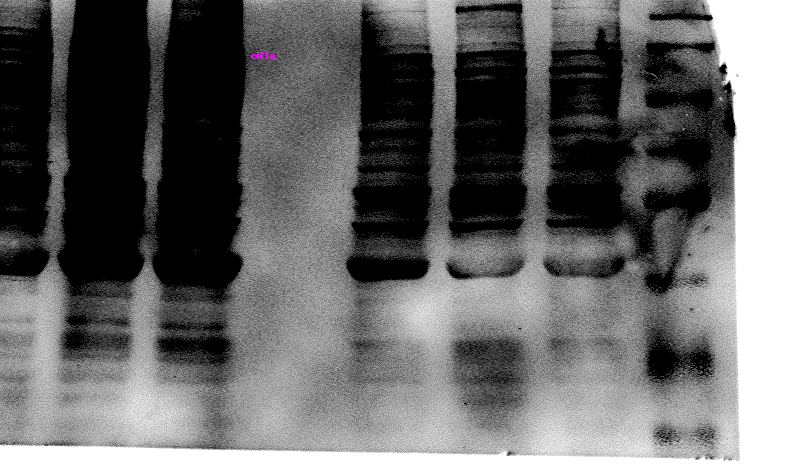
a-sma


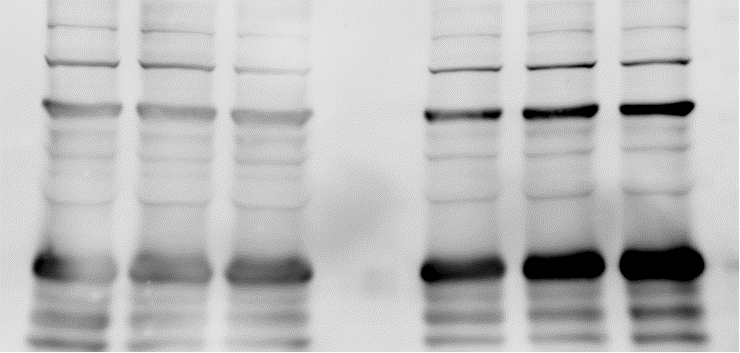


GAPDH

1. Full uncropped Gels and Blots images for Figure2D


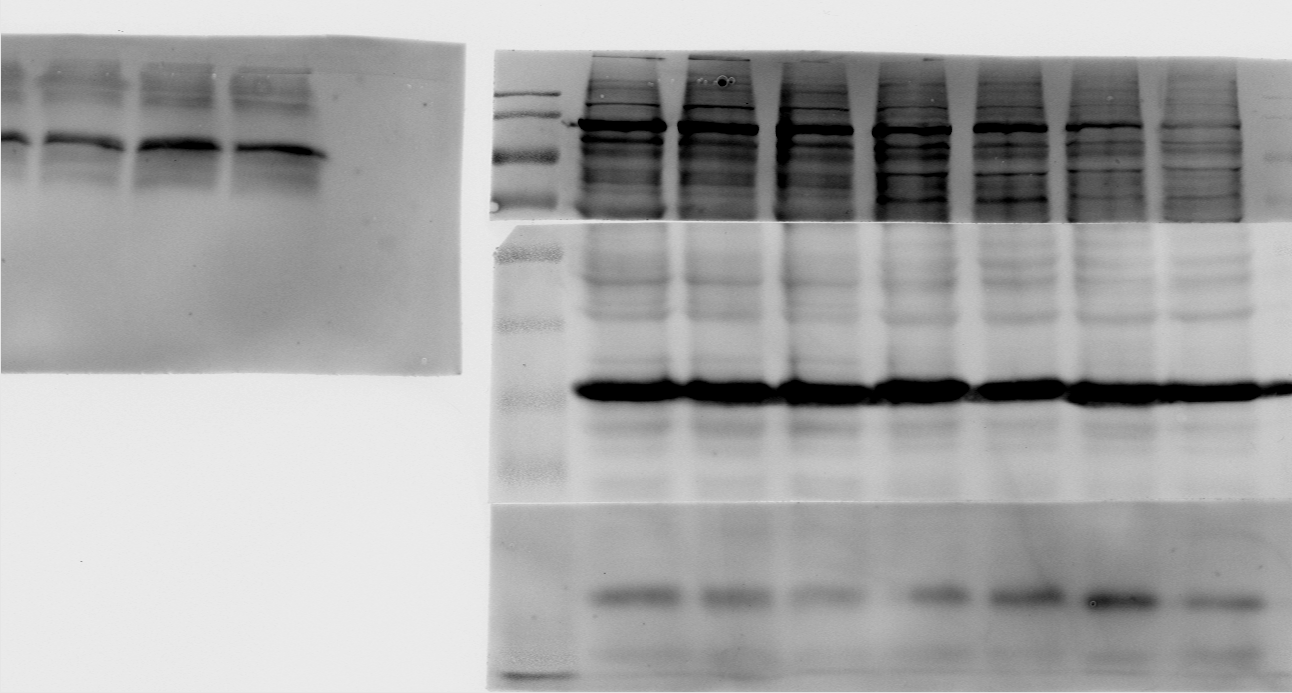
NC circRNA(+)

Col1a1(130KD)

NC circRNA+


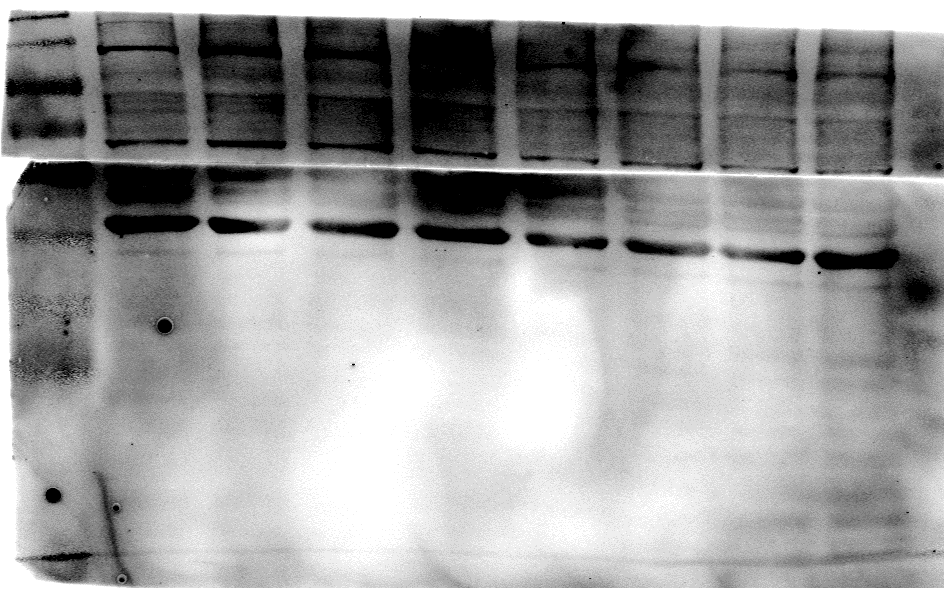


a-SMA(43KD)

NC circRNA+


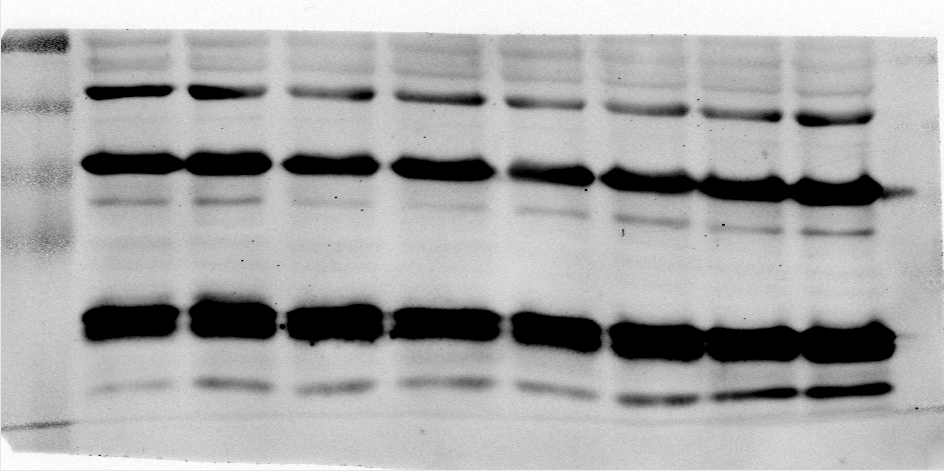


GAPDH(36KD)

1. Full uncropped Gels and Blots images for Figure3H


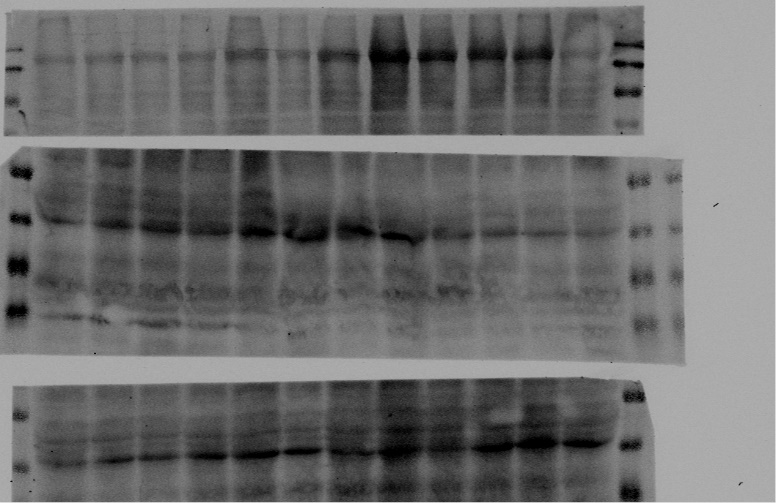


Col1a


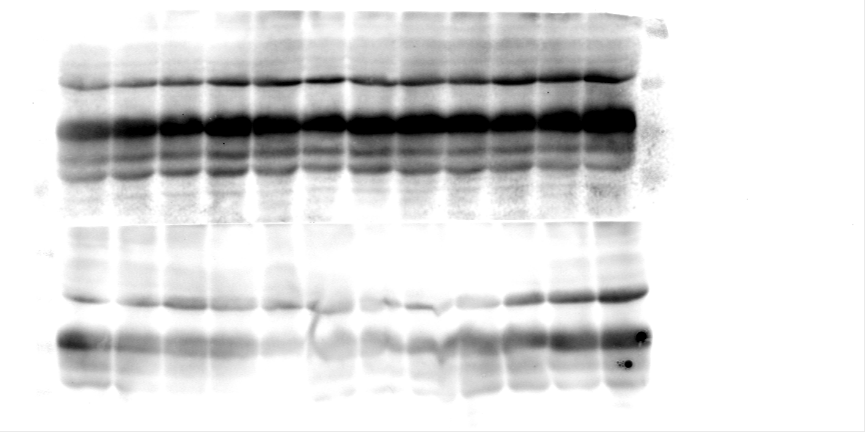


GAPDH


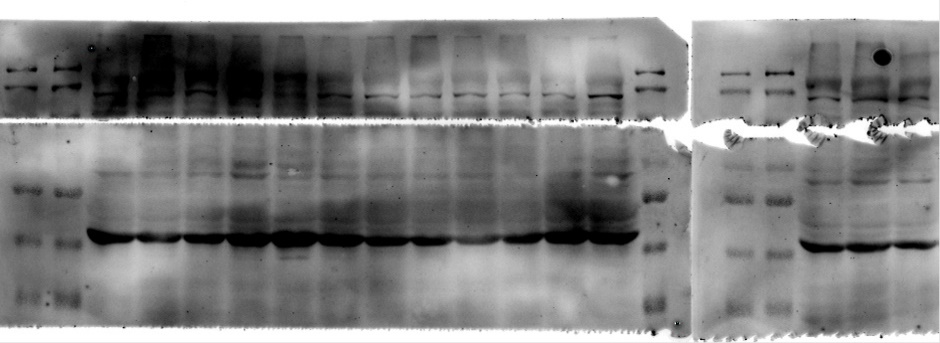


a-sma


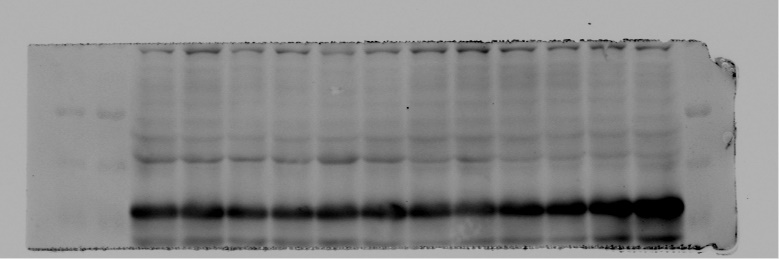


GAPDH

1. Full uncropped Gels and Blots images for Figure6F

Cytoplasm Nucleus total

NC circ NC circ NC circ


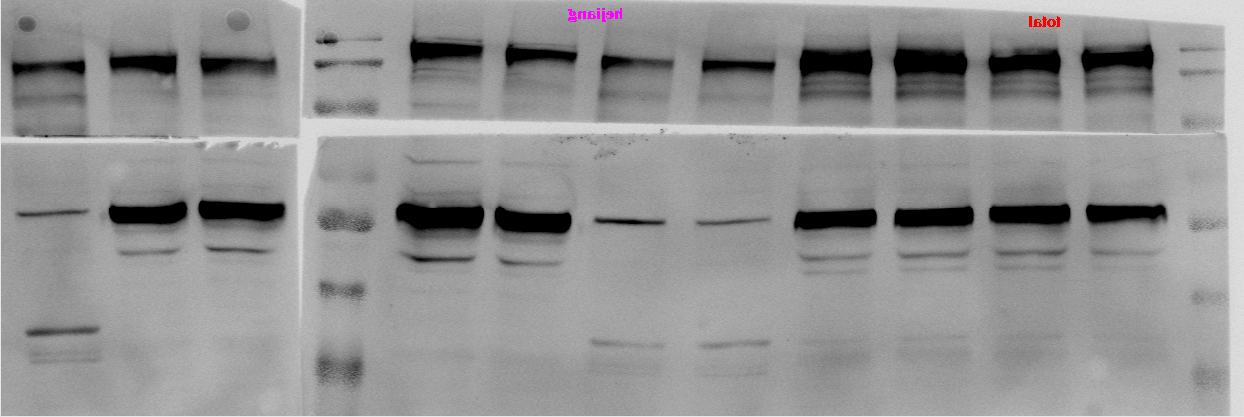


PKM2(60KD)

Cytoplasm Nucleus total


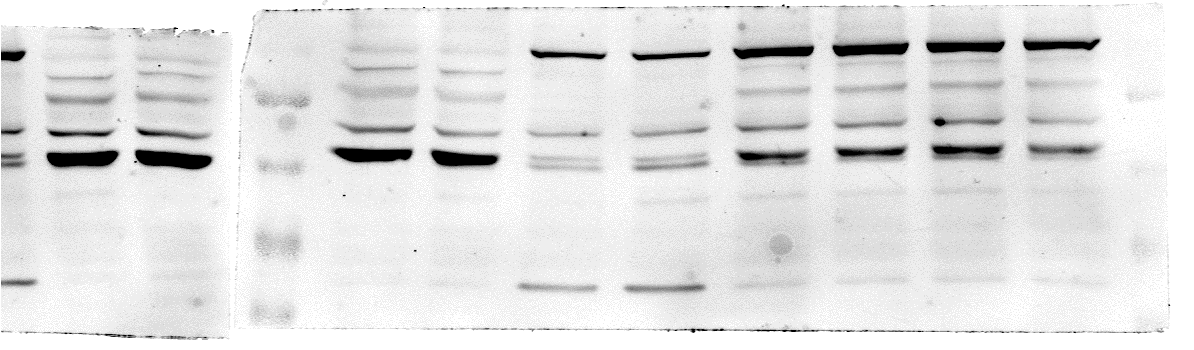
 NC circ NC circ NC circ

LaminB1

Actin

Another repeat

Cytoplasm Nucleus total

NC circ NC circ NC circ


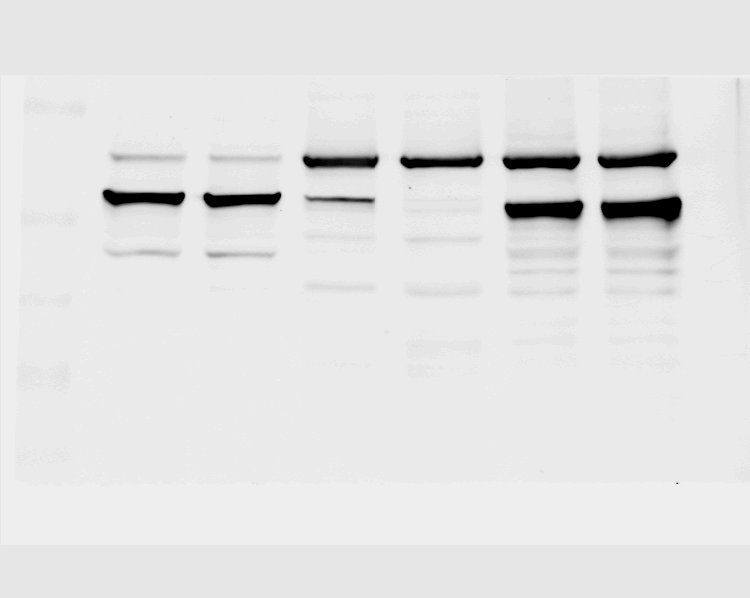


LaminB1

PKM2


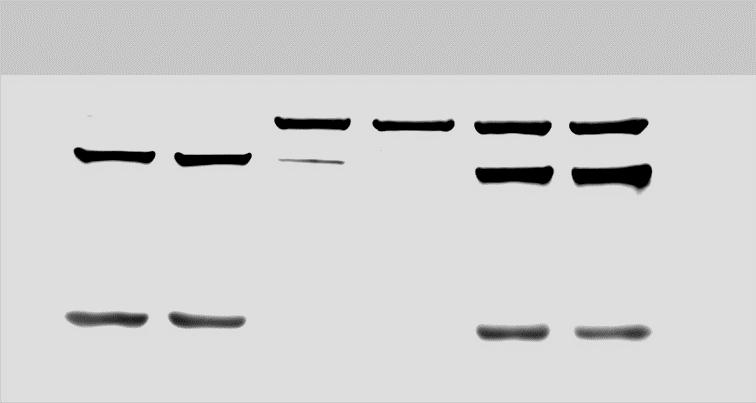


LaminB1

PKM2

GAPDH

1. Full uncropped Gels and Blots images for Figure6G

NC circ+


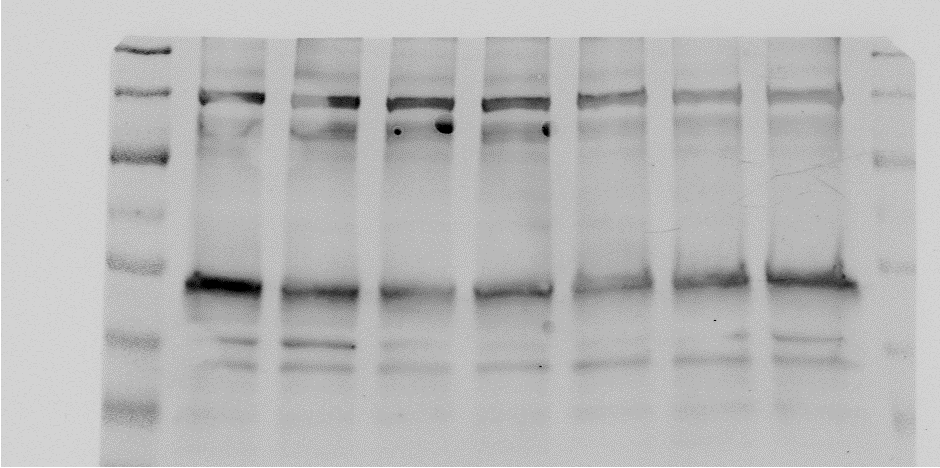


P-Pkm2


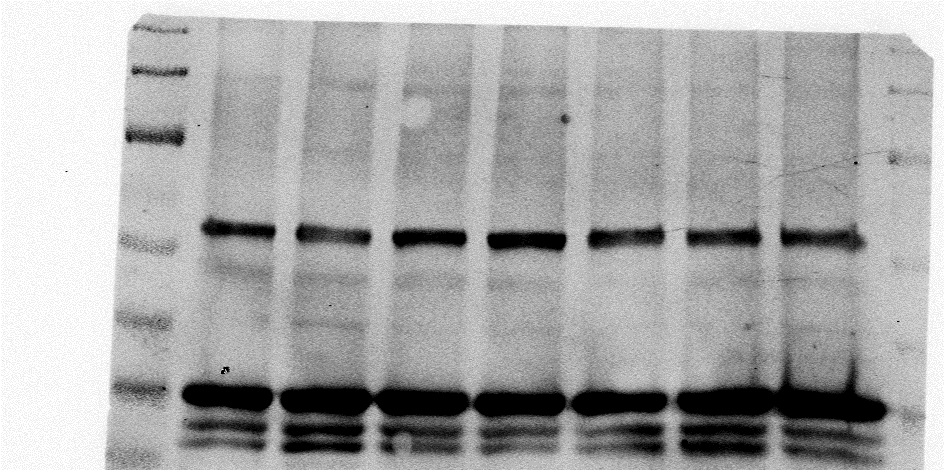


PKM2

GAPDH

1. Full uncropped Gels and Blots images for Supplefigure2B


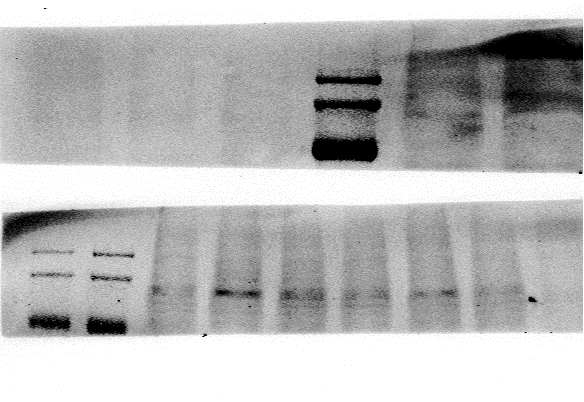
 Circ+ NC circ+

Col1a1

Circ+ NC circ+


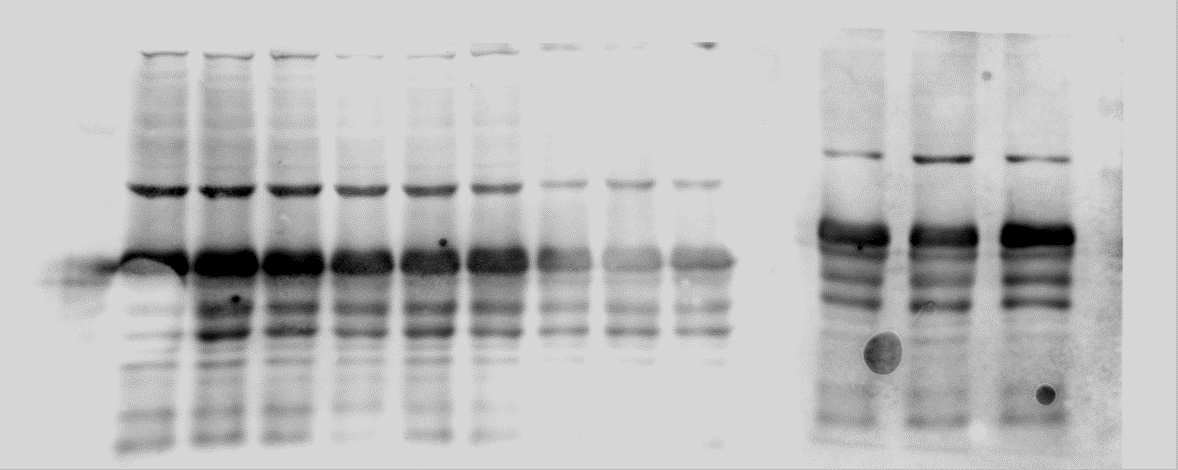


GAPDH

NC circRNA





1. sma



 NC circ+

GAPDH

Another repeat

NC circRNA+


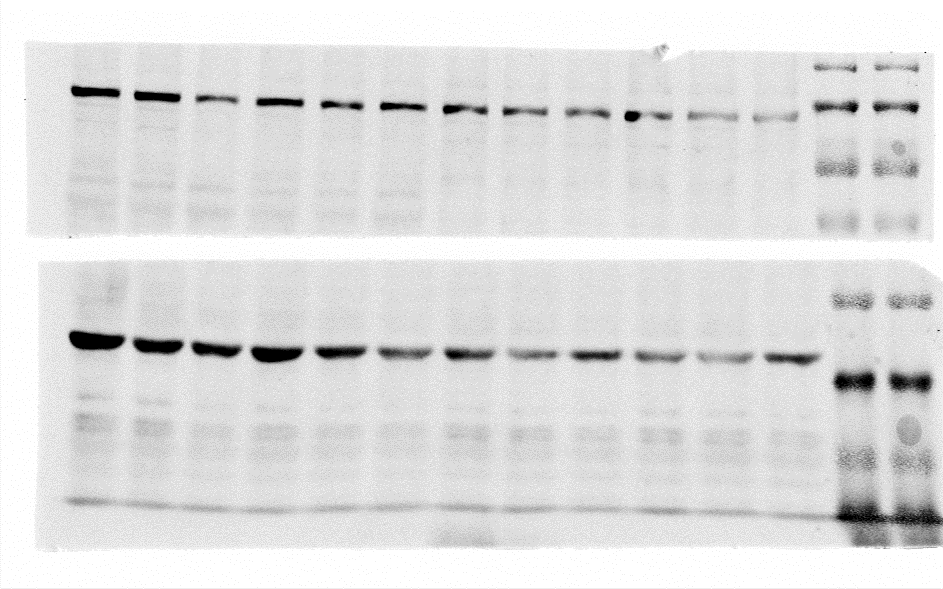


a-sma

NC circRNA+


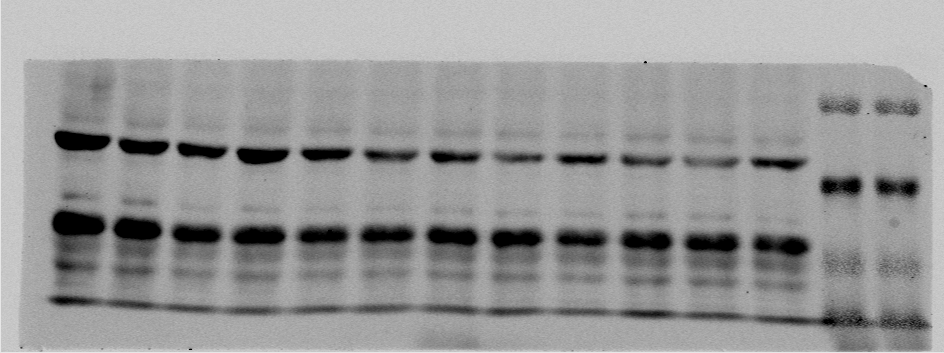


GAPDH
